# Ape parasite origins of human malaria virulence genes

**DOI:** 10.1038/ncomms9368

**Published:** 2015-10-12

**Authors:** Daniel B. Larremore, Sesh A. Sundararaman, Weimin Liu, William R. Proto, Aaron Clauset, Dorothy E. Loy, Sheri Speede, Lindsey J. Plenderleith, Paul M. Sharp, Beatrice H. Hahn, Julian C. Rayner, Caroline O. Buckee

**Affiliations:** 1 Center for Communicable Disease Dynamics, Harvard School of Public Health, Boston, Massachusetts 02115, USA; 2 Department of Epidemiology, Harvard School of Public Health, Boston, Massachusetts 02115, USA; 3 Department of Medicine, Perelman School of Medicine, University of Pennsylvania, Philadelphia, Pennsylvania 19104, USA; 4 Department of Microbiology, Perelman School of Medicine, University of Pennsylvania, Philadelphia, Pennsylvania 19104, USA; 5 Sanger Institute Malaria Programme, The Wellcome Trust Sanger Institute, Hinxton, Cambridge CB10 1SA, UK; 6 Department of Computer Science, University of Colorado, Boulder, Colorado 80309, USA; 7 Santa Fe Institute, Santa Fe, New Mexico 87501, USA; 8 BioFrontiers Institute, University of Colorado, Boulder, Colorado 80303, USA; 9 Sanaga-Yong Chimpanzee Rescue Center, IDA-Africa, Portland, Oregon 97204, USA; 10 Institute of Evolutionary Biology and Centre for Immunity, Infection and Evolution, University of Edinburgh, Edinburgh EH9 3JT, UK

## Abstract

Antigens encoded by the *var* gene family are major virulence factors of the human malaria parasite *Plasmodium falciparum*, exhibiting enormous intra- and interstrain diversity. Here we use network analysis to show that *var* architecture and mosaicism are conserved at multiple levels across the *Laverania* subgenus, based on *var*-like sequences from eight single-species and three multi-species *Plasmodium* infections of wild-living or sanctuary African apes. Using select whole-genome amplification, we also find evidence of multi-domain *var* structure and synteny in *Plasmodium gaboni*, one of the ape *Laverania* species most distantly related to *P. falciparum*, as well as a new class of Duffy-binding-like domains. These findings indicate that the modular genetic architecture and sequence diversity underlying *var*-mediated host-parasite interactions evolved before the radiation of the *Laverania* subgenus, long before the emergence of *P. falciparum.*

## Abstract (web summary)

Antigens encoded by *var* genes are major virulence factors of the human malaria parasite *Plasmodium falciparum*. Here, Larremore *et al*. identify *var*-like genes in distantly related *Plasmodium* species infecting African apes, indicating that these genes already existed in an ancestral ape parasite many millions of years ago.

## Graphical abstract

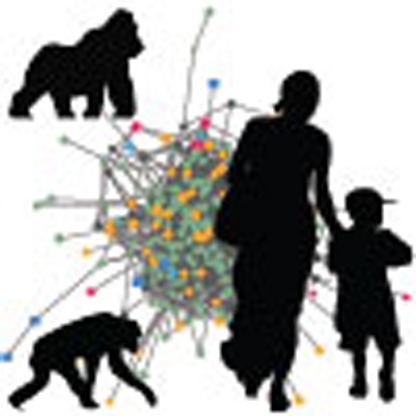

Wild-living apes in Africa are naturally infected by at least six *Plasmodium* species that form a separate subgenus, termed *Laverania*^1^,^2^,^3^,^4^,^5^,^6^,^7^,^8^,^9^,^10^. Three of these species, *P. reichenowi, P. gaboni* and *P. billcollinsi*, have been found only in chimpanzees, while the other three, *P. adleri, P. blacklocki* and *P. praefalciparum*, have been found only in gorillas (Fig. 1a). Zoonotic transfer has occurred at least once, when a gorilla parasite (*P. praefalciparum*) gave rise to human *P. falciparum*, which causes the vast majority of malaria-associated morbidity and mortality in humans^1^,^10^.

A key component of *P. falciparum* virulence is the parasite's ability to cause infected erythrocytes to adhere to the vascular endothelium. This allows the parasite to escape elimination in the spleen but can also lead to vascular obstruction and inflammation, key components of severe pathological complications such as cerebral malaria^11^,^12^. Cytoadherence is mediated by members of the *P. falciparum* erythrocyte membrane protein 1 (PfEMP1) family, which contain between three and eight different Duffy-binding-like (DBLα-ζ) and cysteine-rich interdomain region (CIDRα-δ) domains and are expressed on the surface of infected erythrocytes, where they bind to endothelial receptors. Each *P. falciparum* genome encodes ∼60 different PfEMP1 proteins, which are expressed from *var* genes, one at a time, by means of epigenetic regulation^13^,^14^. Given their central role in *P. falciparum* pathogenesis, but absence from all other human *Plasmodium* species, the origins of *var* genes are of particular interest.

Three factors have limited our ability to investigate the evolutionary history of *var* genes. First, obtaining blood samples from *Laverania*-infected wild-living apes is not ethical. As a result, all ape-derived *var* sequences analysed to date come from a single *P. reichenowi* parasite, called PrCDC, from a wild-born chimpanzee, who was found to be *Plasmodium* infected in captivity^15^. Second, *P. falciparum var* genes are highly diverse (Fig. 1b). Not only is there rapid recombination between genes within and across chromosomes, which shuffles gene content within genome repertoires during infection^16^,^17^, but sexual reproduction in the mosquito vector also generates diversity via reassortment of chromosomes and conversion events^18^. Thus, conventional phylogenetic approaches fail to resolve evolutionary relationships between *var* genes, requiring new and recombination-tolerant analysis techniques^19^,^20^,^21^,^22^,^23^,^24^. Finally, the mosaicism and diversity generated by rapid recombination^16^,^17^, combined with the fact that most *var* genes are subtelomeric, render the assembly of full-length *var* genes from shotgun sequenced parasite genomes extremely difficult^25^,^26^.

Here we overcome these impediments by generating 369 new *var* sequence fragments from five ape *Laverania* species, derived by PCR amplification from faecal and blood samples of naturally infected wild-living and sanctuary apes, respectively. We use network approaches and other recombination-tolerant methods to analyse these new sequences, together with 353 previously reported *var* gene sequences from one *P. reichenowi* and seven *P. falciparum* isolates^15^,^19^. In addition, we identify and analyse partially assembled *var*-like sequences from otherwise near-full-length genomes of two *P. gaboni* parasites (SYpte37 and SYptt75), one of the *Laverania* species most distantly related to *P. falciparum*^27^,^28^. Analysis of these sequences reveals that several PfEMP1 domains, as well as the genetic structure and multi-domain architecture that are characteristic of *P. falciparum var* genes, are present across the *Laverania* subgenus. Thus, many *var* multi-gene family features predate the most recent common ancestor of extant *Laverania* species.

## Results

### *Laverania* species identification and sequence generation

To study *var* gene architecture in ape *Laverania* species, we first determined the *Plasmodium* species composition of 11 blood and faecal samples from sanctuary and wild-living apes using a limiting dilution PCR approach called single-genome sequencing (SGS)^29^. To ensure amplification of single-parasite templates, blood and faecal DNA was diluted such that <30% of all PCR reactions yielded an amplification product. Amplicons were sequenced directly without cloning into a plasmid vector and sequences containing ambiguous bases indicative of template mixtures were discarded. This approach eliminates *Taq* polymerase-induced recombination (template switching) and nucleotide misincorporations in finished sequences, and also ensures a proportional representation of plasmodial variants as they exist *in vivo* (see the Methods section for a more detailed description of SGS). Targeting eight different mitochondrial, apicoplast and nuclear loci and sequencing up to 174 different SGS amplicons per sample (Supplementary Table 1), we identified eight samples with single-species infections of *P. reichenowi* (C1), *P. gaboni* (C2), *P. billcollinsi* (C3) or *P. praefalciparum* (G1). Three additional faecal samples represented mixed-species infections of several gorilla or chimpanzee parasites, including one of unknown, non-*Laverania* species origin (Supplementary Table 1).

**Figure 1:**
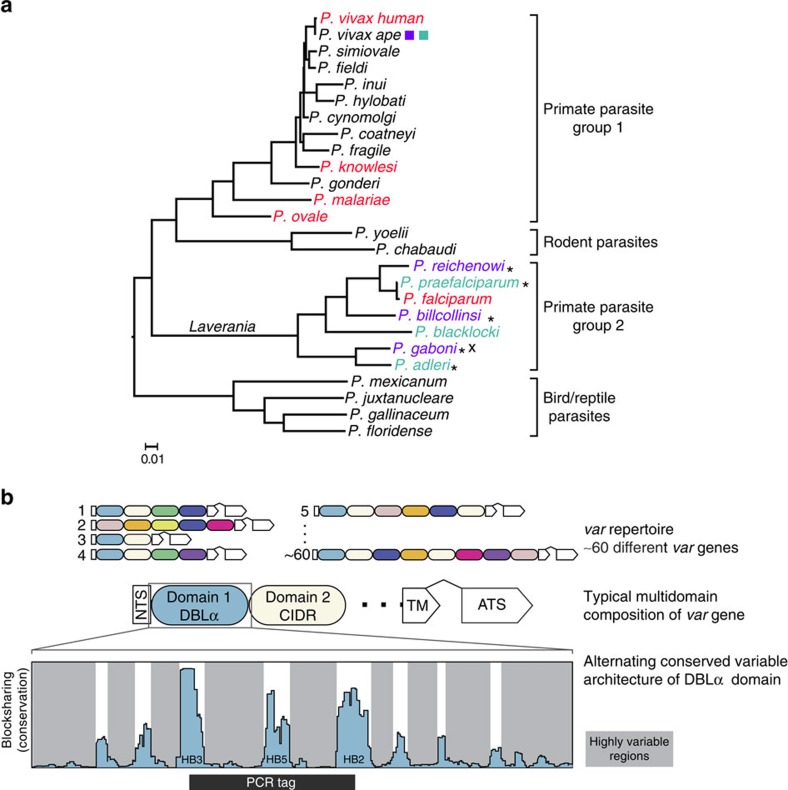
Characterization of *Laverania var* gene sequences. (**a**) Phylogeny of *Plasmodium* species. The tree was constructed from mitochondrial sequences (2.4-kb spanning *cox*1 and *cyt*B). The scale bar indicates 0.01 substitutions per site. Colours indicate species infecting humans (red), chimpanzees (purple) and gorillas (aqua). Asterisks indicate successful PCR amplification of *var* sequences; a cross indicates identification of *var*-like genes in near-full-length *P. gaboni* genomes. (**b**) Three-level schematic of modular *var* diversity, structure and architecture. Coloured ovals represent classes of DBL or CIDR domains. White boxes represent the N-terminal segment (NTS), transmembrane (TM) and acidic terminal segment (ATS) domains; a wedge between TM and ATS domains indicates the intron that separates the two *var* exons. Alternating conserved variable architecture is illustrated using blocksharing (see the Methods section) between one representative DBLα domain (DD2var11) and other DBLα domains published by Rask *et al*^19^. A black bar indicates the location of the PCR amplified DBLα tag region, which spans three conserved homology blocks (HB3, HB5 and HB2)^19^, 72–147 amino acids in length.

**Figure 2:**
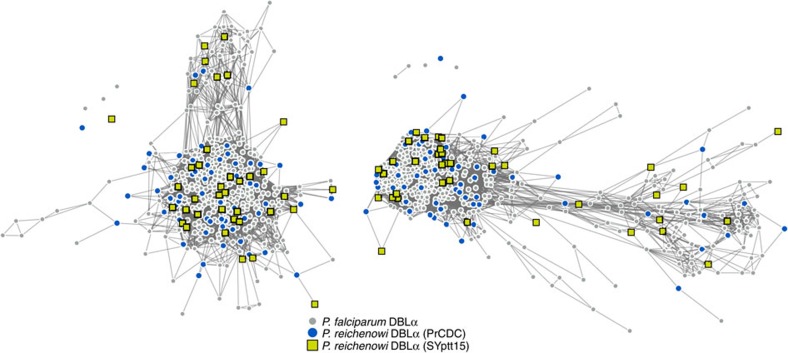
Networks of DBLα sequences from *P. reichenowi* and *P. falciparum*. Each node represents a DBLα HVR sequence and each link represents a shared amino-acid substring of significant length^21^. *Laverania* species and strain origin is indicated by node colour and shape. Left and right networks correspond to left and right HVRs, respectively. *P. falciparum* and *P. reichenowi* sequences do not cluster by species or sample in either HVR. Link lengths and node placements are determined by a force-directed layout to better reveal structure, if it exists (see the Methods section). Additional analyses of these networks are shown in Supplementary Fig. 1.

Given their enormous diversity, *var* homologs were amplified targeting a conserved region of the DBLα domain, termed the *var* gene ‘tag', using conventional PCR and previously reported primers^30^,^31^ (see the Methods section and Supplementary Table 2). Amplicons were cloned, and multiple clones per sample were sequenced and grouped into unique haplotypes by phylogenetic analysis. The *var* gene tag is commonly analysed because it is sufficiently conserved in two locations to allow reliable amplification, and is located within the DBLα domain, which, unlike other DBL domains, is present in almost all *var* genes^20^,^21^,^22^,^30^,^31^,^32^. The DBLα tag consists of three conserved homology blocks^19^ (HBs) interspersed with highly variable regions (HVRs) of diverse length and sequence content (Fig. 1b), an architecture that facilitates mosaicism^21^. Standard sequence analysis techniques cannot adequately analyse these mosaic sequences^19^,^20^,^21^,^22^,^23^,^24^ and we therefore used a network analysis method to characterize the evolutionary relationships between *Laverania var* fragments. Figure 2 illustrates this type of analysis, where each node represents a *var* DBL sequence tag and a link between two nodes represents a shared identical sequence mosaic element. Due to frequent recombination and the possibility that immune selection differs between adjacent HVRs, networks were constructed independently for each of the two HVRs, which in *P. falciparum* were shown to exhibit different community structures^21^. For each sample, only unique *var* tag haplotypes were included into the analysis (see the Methods section for a detailed description of network construction and statistical community detection).

### Shared *var* mosaic structure in *P. reichenowi* and *P. falciparum*

We first examined the 37 new DBLα tags from a *P. reichenowi* monoinfection detected by routine blood analysis in an asymptomatic sanctuary chimpanzee (SYptt15), who was housed in close proximity to the habitat of wild apes. It is well established that human *P. falciparum* and chimpanzee *P. reichenowi* are closely related sister taxa^15^, and previous analyses of PrCDC *var* gene sequences indicated sequence homology with field and lab strains of *P. falciparum*^15^,^20^,^22^,^23^,^33^. While early studies investigated shared polymorphisms in preliminary assemblies of a small subset of these genes^20^, more recent studies analysed the complete set of PrCDC DBLα domains, finding conserved gene regions between PrCDC and *P. falciparum* isolates 3D7 and HB3 (ref. 23), as well as the presence of *P. falciparum* HBs in PrCDC DBLα sequences^33^. In contrast, we focused specifically on the most polymorphic HVR regions of *P. falciparum* and *P. reichenowi* DBLα homologs. Using a network community detection algorithm, a Bayesian *k*-mer analysis and a pairwise distance approach, we found that *var* mosaics within the *P. falciparum–P. reichenowi* network do not cluster by parasite species (Fig. 2; Supplementary Fig. 1a,b), and that *var* genes from both species exhibit the same modular HVR architecture, that is, a pattern of alternating regions of conservation and variability (Supplementary Fig. 1c). We have previously hypothesized that this genetic structure may allow for neighbouring HVRs to respond independently to different selection pressures^21^. Thus, our results confirm and extend previous findings that DBLα organization and capacity for diversification in response to immune selection were already present in the most recent common ancestor of *P. falciparum* and *P. reichenowi.*

### *var* DBLα tag structures predate the *Laverania* radiation

**Figure 3:**
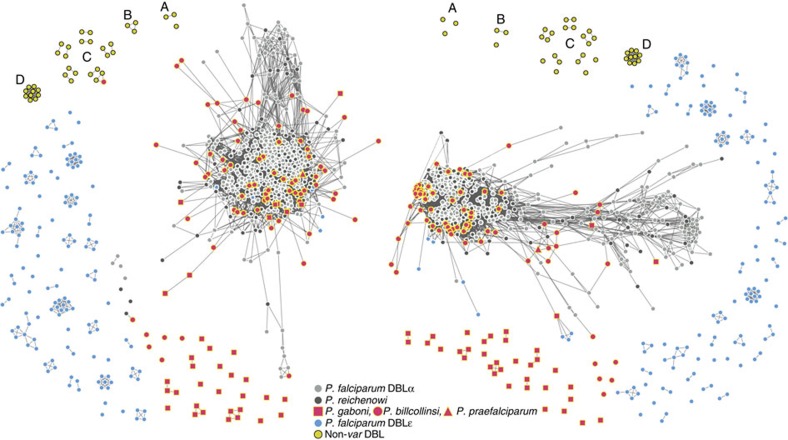
Networks of DBL sequences from *Laverania* single-species infections in the context of known DBLα and non-DBLα sequences. Each node represents a DBL HVR sequence from a single-species infection and each link represents a shared amino-acid substring of significant length. Note that for each sample, only unique *var* DBL haplotypes were included in the network analysis. Nodes with zero links indicate sequences that share no significant amino-acid substrings with other sequences. Networks were built separately for each HVR, where mosaic diversity is highest (see the Methods section). Colours correspond to *Laverania* species as indicated; annotated yellow nodes correspond to (A) *dblsmsp*1 and (B) *dblmsp*2 from Pf3D7, PfIT and PrCDC; (C) both DBL domains from *ebl1*, *eba140*, *eba165*, *eba175* and *eba181* of Pf3D7 and PfIT; (D) *P. vivax* Duffy-binding proteins; see Supplementary Table 3 for a comprehensive list of non-DBLα sequences.

Having analysed *var* tags from *P. falciparum* and *P. reichenowi*, we next examined parasite sequences from across the ape *Laverania* subgenus. Numerous identical mosaic elements in otherwise divergent sequences and a shared overall HVR architecture extended to the most divergent species (Fig. 3; Supplementary Fig. 2). We were able to reconstruct highly connected networks for each HVR, indicating the presence of shared mosaic elements among the vast majority of tags from single-species parasite infections. Every *Laverania var* tag contained three conserved sequence motifs separating two HVRs: in 86% of sequences, the three conserved motifs corresponded to three of the five most common *P. falciparum var* motifs (in the order: HB3, HB5 and HB2)^19^, while in the remaining 14%, HB5 was intact in the middle of the tag and more divergent forms of HB3 and HB2 were encoded by the 5′ and 3′ end of the tag, respectively (Supplementary Fig. 3).

We confirmed that these tags were not derived from non-*var* DBL-containing genes by including tags from *P. falciparum* erythrocyte-binding antigen (*eba*) genes, *P. falciparum* and *P. reichenowi* DBL merozoite surface protein 1 (*msp3.4*) and DBLMSP2 (*msp3.8*), and *P. vivax* Duffy-binding proteins in our analysis (Supplementary Table 3). We also included *P. falciparum* DBLɛ tags to compare tags with *var*-derived, yet non-DBLα, sequences. As shown in Fig. 3, tags from single-species ape *Laverania* infections remained separated from both the non-*var* DBL tags and the *P. falciparum* DBLɛ tags, with a majority connected to one or both of the large connected components formed by the *P. falciparum* and known *P. reichenowi* tags. This majority included every new *P. reichenowi* and *P. praefalciparum* tag, and all but one *P. billcollinsi* tag. On the other hand, only 10 *P. gaboni* tags were connected to one or both large components, with the other 26 connected only to other *P. gaboni* tags in separate, small components. These smaller *P. gaboni* components did not share mosaic elements with DBLɛ or non-*var* DBL sequences, suggesting that they represented divergent, yet *var*-like, domains.

### *Laverania* parasites contain ape-specific *var*-like DBL domains

**Figure 4:**
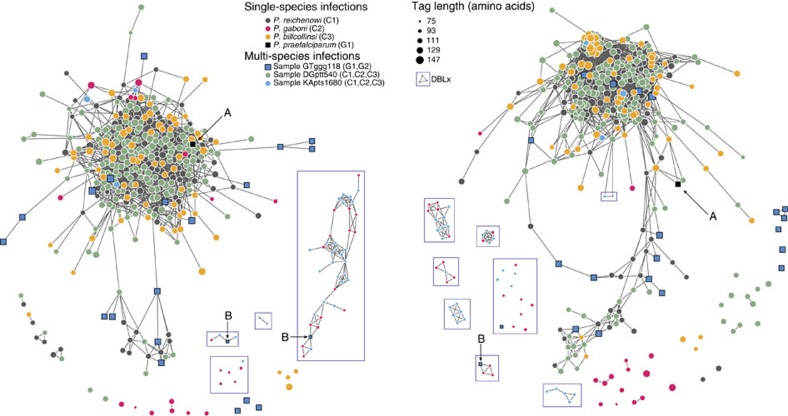
Networks of DBL sequences from single- and multi-species *Laverania* infections. Each node represents a DBL HVR sequence and each link represents a shared amino-acid substring of significant length. Note that for each sample only unique *var* DBL haplotypes were included in the network analysis. Nodes with zero links indicate sequences that share no significant amino-acid substrings with other sequences. Networks were built separately for each HVR, where mosaic diversity is highest (see the Methods section). Circular nodes represent chimpanzee parasites and square nodes represent gorilla parasites. Node colour corresponds to species and node size corresponds to tag length as indicated. DBLx sequences are enclosed in boxes. Annotations call attention to (A) *P. praefalciparum* single-species infection sequence; (B) DBLx sequences from gorilla samples, hypothesized to be *P. adleri*, that share mosaic elements with DBLx chimpanzee parasites.

We next investigated the relationships between sequences from all ape *Laverania* samples by conducting a network analysis that excluded *P. falciparum*, but included sequences from both mixed-species and single-species infections (Fig. 4). Sequences from *P. billcollinsi* and *P. praefalciparum* remained integrated within the large connected component that also included *P. reichenowi*, indicating conservation of mosaic elements within HVRs across these species. This finding is consistent with mitochondrial DNA (Fig. 1a), apicoplast and nuclear phylogenies^1^,^34^, which place *P. billcollinsi* and *P. praefalciparum* closer to *P. reichenowi*. In contrast, sequences from four single-species infections of *P. gaboni*, which represent a much more distant *Laverania* species, exhibited much less shared sequence content in HVR networks. However, *P. gaboni* sequences appeared to fall into two subgroups based on tag length: (i) longer *P. gaboni* sequences (94-135 amino acids), which share mosaic elements with *P. reichenowi* and *P. billcollinsi* in 8 of 15 sequences in the left HVR and 2 of 15 sequences in the right HVR, and which we therefore term DBLα-like (red, unboxed in Fig. 4); and (ii) shorter *P. gaboni* sequences (72-85 amino acids), which remain disconnected from the *P. reichenowi–P. billcollinsi* component in 21 of 21 cases and which we therefore termed DBLx-like (red, boxed in Fig. 4). Thus, within the HVRs, longer *P. gaboni* DBLα-like sequences are partially overlapping with *P. reichenowi* and *P. billcollinsi*, while the shorter sequences appear to be distinct.

Although the DBLx tags fell outside the large connected component of the *P. reichenowi*-*P. billcollinsi* network (Fig. 4, boxes), they were all amplified using standard *P. falciparum* DBLα primers, and they all exhibited the classical DBL architecture with fully intact HB5 motifs in the tag centre. However, they were unrelated to other known DBL domain classes (Supplementary Fig. 4). All four single-species *P. gaboni* samples, as well as one *P. gaboni*-containing mixed-species sample, contained DBLx sequences. On the basis of polymorphisms in the HB3-like region, DBLx sequences formed two subgroups, which we refer to as DBLx1 and DBLx2 (Supplementary Fig. 3; see the Methods section). DBLx sequences were not limited to chimpanzee parasites, as the mixed-species infection gorilla sample GTggg118, which contained both *P. praefalciparum* and *P. adleri*, also featured DBLx2 tags. The GTggg118 DBLx2 tags shared mosaic elements with both DBLx1 and DBLx2 tags from *P. gaboni*, while the GTggg118 DBLα-like tags were well-connected to the *P. billcollinsi-P. reichenowi* component (Fig. 4). We thus hypothesize that the GTggg118 DBLx2 tags derive from *P. adleri*, a closely related sister taxon to *P. gaboni* (Fig. 1a), while the DBLα-like tags may be derived from either *P. adleri* or *P. praefalciparum*. Thus, it is likely that DBLx sequences represent new *var*-like DBL subdomains that are restricted to the C2/G2 branch of the *Laverania* subgenus (Fig. 1a).

### *var* multi-domain structures predate the *Laverania* radiation

To confirm the presence of *var*-like genes in *P. gaboni*, we also examined near-full-length parasite genomes and unplaced contigs, which were derived by select whole-genome amplification^27^,^28^ from two chimpanzee blood samples (SYpte37 and SYptt75). Three lines of evidence indicated that *var*-like genes, consisting of multiple DBL domains, were indeed present in this parasite species. First, we identified 55 *var*-like DBL domains in 40 different contigs, 14 and 2 of which were further classified using the VarDom server^19^ as being related to *P. falciparum* DBLɛ and DBLζ domains, respectively (Table 1; Methods). None of the remaining DBL domains could be similarly subclassified, but four contigs featured exact nucleotide matches for DBLα-like tag sequences, providing a cross-validation between methods. Three contigs featured three, four, and five adjacent and non-identical DBL domains, a configuration unique to *vars*. An additional six contigs featured two adjacent DBL domains, but in these cases an *eba* gene origin could not formally be excluded^35^.

**Figure 5:**
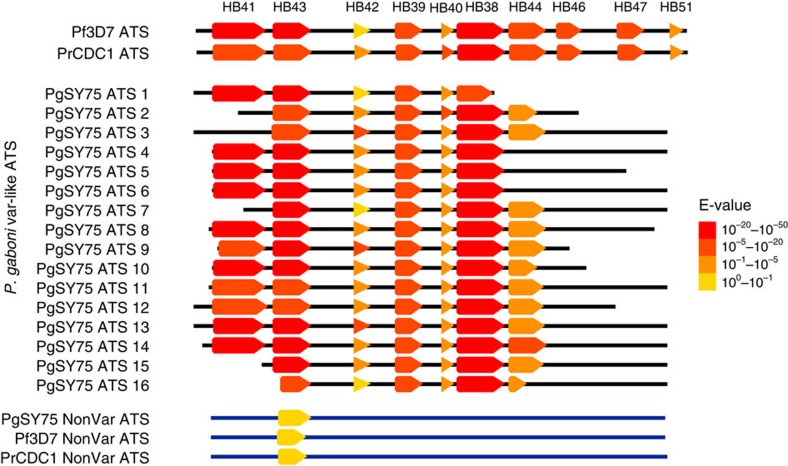
Conservation of *var* ATS domain homology block structure in *P. gaboni*. The homology block (HB) structure of *var* ATS domains identified in 16 contigs of a near complete *P. gaboni* genome (PgSY75) are shown in relation to representative *P. falciparum* and *P. reichenowi var* ATS domains (Pf3D7 and PrCDC1, top) as well as a non-*var “*ATS-like” domain of the *P. falciparum* PF3D7_0113800 gene and its *P. reichenowi* and *P. gaboni* orthologues (bottom). HBs (arrows) were predicted by VarDom 1.0 and annotated in an alignment of all 20 sequences. Colours correspond to VarDom reported E-values, representing an estimate of the likelihood of observing such a match by random chance. Black lines indicate the relative length of each sequence.

The finding of only nine contigs with *var*-like multi-DBL configurations in our *P. gaboni* genomic data is likely related to difficulties in assembling these sequences from short read data. *De novo* assembly is hindered by identical and near-identical motifs present in different DBL domains, which make an accurate determination of the number and order of these domains in a given *var* gene difficult^36^. In contrast, acidic terminal segment (ATS) domains, which are also a unique feature of *var* genes, lack these repeat structures, although they share some sequence motifs due to frequent recombination^37^. We thus reasoned that ATS regions would more likely assemble into full-length or near-full-length domains and looked for these *var* signatures in the *P. gaboni* genomic sequences. Indeed, ATS domains were readily identified in 16 contigs derived from the *P. gaboni* SYptt75 genome. In *P. falciparum*, the ATS domain encodes the intracellular portion of the PfEMP1 protein, which is expressed from a separate exon (Fig. 1b). ATS domains are unique to *var* genes, except for a single-copy non-*var* gene with an ‘ATS-like' domain on chromosome 1 (PF3D7_0113800)^19^. Using the VarDom server to characterize the *P. gaboni* ATS domains, we identified seven of ten known major HBs (Fig. 5). These were very similar to *P. falciparum var* ATS HBs, but very different from the non-var ‘ATS-like' domains of PF3D7_0113800 and its *P. reichenowi*, and *P. gaboni* orthologs (Fig. 5; Supplementary Fig. 5), thus providing compelling evidence for the presence of bona fide *var* ATS domains in *P. gaboni*.

**Figure 6:**
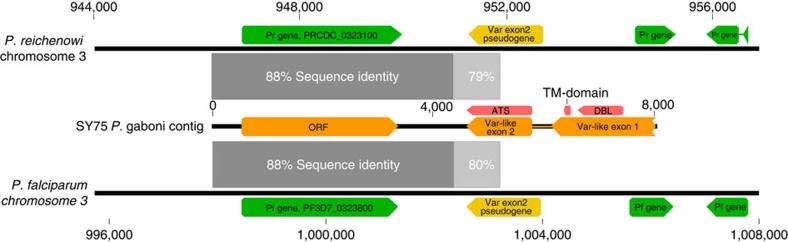
Shared synteny of *var*-like genes in *P. falciparum*, *P. reichenowi* and *P. gaboni*. An open-reading frame (ORF) located downstream of a predicted *var*-like gene in *P. gaboni* showed 88% sequence identity (dark grey bars) with a single-copy gene present in both *P. falciparum* 3D7 (PF3D7_0323800) and *P. reichenowi* CDC1 (PRCDC_0323100). The *P. gaboni var*-like gene is syntenic with a *var* exon 2 pseudogene in both *P. falciparum* and *P. reichenowi*, suggesting that a *var* gene was present at this location in the ancestor of all three *Laverania* species.

Finally, three of the ATS-containing contigs exhibited a longer two-exon *var* gene structure, with a DBL and transmembrane domain in exon 1 and an ATS domain in exon 2. One of these contigs contained an additional open-reading frame (ORF) downstream of the *var*-like exon 2, which was 88% identical in its nucleotide sequence to genes and intergenic flanking sequences in *P. falciparum* (PF3D7_0323800) and *P. reichenowi (*PRCDC_0323100) on the same chromosome, respectively (the latter two shared 94% nucleotide sequence identity). Although the function of these orthologs is unknown, they are single-copy genes immediately adjacent to a *var* exon 2 pseudogene on chromosome 3 of both *P. falciparum* and *P. reichenowi* (Fig. 6). This synteny implies the existence of ancestral ORFs on chromosome 3, including a *var* gene that retained both exons in *P. gaboni*, but represents a single-exon pseudogene in *P. falciparum* and *P. reichenowi*. Thus, the presence of a two-exon *var* structure and synteny on chromosome 3 for three *Laverania* species, which span the root of the subgenus phylogeny, indicate that *var* genes evolved their extant two-exon and multi-domain structure before the radiation of this subgenus.

### *Laverania var* repertoire structure

It has previously been shown that *P. falciparum var* genes can be divided on the basis of DBLα domains into two main groups, classified by the number of cysteine residues in the tag region^30^, which map to distinct community structures in network analyses^21^. These two main groups can be further subdivided into a total of six Cys/PoLV (CP) groups based on the presence or absence of key amino acid residues^30^,^38^. These cysteine-based classifications were found to be associated with different upstream promoter regions and clinical outcomes, and *var* repertoires in individual *P. falciparum* parasites appear to be stably structured with respect to these categories^32^. We observed the same cysteine-based organization, both with respect to cysteine counts and CP groups, in DBLα tags from *P. billcollinsi*, but not from *P. gaboni*, although in the latter case we identified far fewer DBLα-like motifs (Supplementary Fig. 6). Thus, cysteine-based organization of *var* gene repertoires extends to *P. billcollinsi*, but may not extend to *P. gaboni* (and by inference *P adleri*).

## Discussion

Until recently, the only known close relative of *P. falciparum* was *P. reichenowi*, a *Laverania* parasite infecting chimpanzees. Over the past 5–6 years, five additional species within the *Laverania* subgenus have been described, each infecting either chimpanzees or gorillas. This *Laverania* species diversity provides an unprecedented opportunity to study the origins of genomic features that previously seemed unique to *P. falciparum*, such as the *var* gene family encoding erythrocyte membrane proteins. Here we show that various aspects of the multi-scale modularity of these loci can be recognized in diverse *Laverania* species, with the implication that a *var* or *var*-like gene family already existed in their last common ancestor. First, at the *var* gene repertoire level, we find genes with a characteristic two-exon structure, encoding multiple adjacent domains potentially capable of binding diverse endothelial markers. Like the constituent domains of the *P. falciparum*-encoded PfEMP1 proteins, the other *Laverania* DBL sequences can be subclassified into distinct groups, which may reflect differences in endothelial binding or other specificities. Second, at the domain architecture level, alternating conserved and hypervariable regions enable combinatorial diversity while presumably maintaining protein structure and binding functions. Finally, at the microscale level, some protein motifs within hypervariable regions are shared among even the most divergent *Laverania* species, despite the evidence of high-frequency recombination within species. Thus, many key elements of the *var* multi-gene family appear to have originated many (perhaps tens of) millions of years ago.

In *P. falciparum*, the *var*-encoded PfEMP1 proteins play a key role in pathogenesis by mediating the binding of infected red blood cells to specific host receptors in a wide range of tissues. Particular disease syndromes have been associated with individual DBL domains, two of which were present in *P. gaboni*. The first, DBLɛ, is found in the *var2csa* genes of *P. falciparum*^19^ and *P. reichenowi*^15^, which exist as only one or two *var* variants per genome and have been identified in every complete *var* repertoire analysed to date. In *P. falciparum*, *var2csa* genes are responsible for placental binding, and the DBLɛ domain has thus been implicated in pregnancy-associated malaria^39^. Similarly, we identified DBLζ in *P. gaboni*. Although there currently are no host receptors or disease syndromes that have been associated with this individual domain in *P. falciparum*, triplet combinations of DBLɛ and DBLζ domains have been linked to IgM-positive rosetting phenotypes^40^. The presence of recognizable DBLɛ and DBLζ domains in the most divergent *Laverania* species suggests that DBL domain differentiation into subtypes represents an ancient host adaptation, and that DBLɛ and DBLζ may represent functionally constrained domains across the *Laverania* subgenus.

Beyond single *var* domains, the *var* repertoires of *P. falciparum* parasites can be divided into groups that have been associated with different clinical phenotypes, such as severe malarial anaemia and cerebral malaria, using a cysteine-based classification of DBLα tags^38^,^41^,^42^. These groups are represented in similar proportions across *P. falciparum* and *P. reichenowi* parasites, and our data suggest that this repertoire structure may also extend to *P. billcollinsi* (Supplementary Fig. 6); an insufficient number of DBLα-like tags precludes an extension of this classification to *P. gaboni* at the present time. Given their association with clinical disease in humans, the extent to which these sequence features are also indicative of pathology in apes warrants further study.

Although we identified *var*-like features in species spanning the *Laverania* subgenus, we also found that certain signatures identified in *P. falciparum* and *P. reichenowi var* genes are absent from the more divergent parasite species. For example, we found no evidence of CIDR domains in either of the *P. gaboni* genomes, despite identifying numerous DBL domains (Table 1). Moreover, DBLα-like *P. gaboni* sequences were not sufficiently similar to *P. falciparum* DBLα domains to be confidently classified as such. Since the vast majority of *P. falciparum var* genes encode a DBLα-CIDR domain pair, the apparent absence of CIDR domains from *P. gaboni* is puzzling, especially in light of the role that CIDR domains are believed to play in host receptor binding^43^. It will be important to determine whether *P. gaboni var*-like genes contain other domains with CIDR-like function or whether *P. gaboni* differs in its biology from other *Laverania* parasites. Second, the network analysis of PCR tags revealed new DBL domains that we termed DBLx because they are unlike the other six known *var* DBL domain classes shared by *P. falciparum*^44^ and *P. reichenowi*^15^ (Fig. 4; Supplementary Figs 3 and 4). These DBLx tags, which were amplified using *P. falciparum* DBLα primers, are shorter than all other tags, and can be further subdivided into DBLx1 and DBLx2 subgroups based on differences in the highly conserved HB3-like region (Supplementary Fig. 3). Divergence from the *P. falciparum* ‘LARSFADIG' motif within this HB3-like region has also been reported for another partially characterized *P. gaboni* genome^15^, but adjacent sequences were not analysed, thus leaving their relationship with DBLx domains unknown. Finally, we identified multiple copies of *P. gaboni* ATS domains, which exhibit a *var*-like HB structure that is very similar, but not identical, to *P. falciparum* and *P. reichenowi* ATS domains (Fig. 5; Table 1; Supplementary Fig. 5). Taken together, these data indicate that, while *var*-like genes in *P. gaboni* (and possibly also *P. adleri*) share important structural similarities with those of *P. falciparum and P. reichenowi*, they also exhibit important differences, which may reflect differences in function and biology.

The presence of *var*-like genes throughout the *Laverania* subgenus suggests an ancient adaptation for antigenic variation, and potentially cytoadherence. However, while links exist between *var* expression and clinical disease in humans, the disease causing potential of *var*-like gene products in *Laverania* parasites infecting wild apes remains unknown. Nonetheless, there may be important parallels since recent field studies of habituated chimpanzees in the Tai Forest, Côte d'Ivoire revealed higher faecal parasite burdens in both young^45^ and pregnant^46^ individuals, similar to what has been described in humans. Given the role of the *var*-encoded PfEMP1 proteins to mediate endothelial binding in the presence of a vigorous host immune response, it is likely that *var* genes play a similar role in other *Laverania* species. However, the extent of *var* gene diversity, especially among the more divergent *Laverania* species that lack certain *P. falciparum*-specific DBL and CIDR domains, suggests potentially different biological solutions. Additional field studies of habituated ape populations will be necessary to establish the biological consequences of ape *Laverania* infections and the pathogenic potential of their *var*-like gene products.

## Methods

### Sample collection

Ape faecal samples were collected from wild-living central (*Pan troglodytes troglodytes*; DGptt540) and eastern (*P. t. schweinfurthii*; KApts1680) chimpanzees and western lowland gorillas (*Gorilla gorilla gorilla*; GTggg140, GTggg118) for previous molecular epidemiological studies of *Laverania* parasites^1^. Samples were collected in RNAlater (1:1 vol/vol), transported at ambient temperatures and stored at −80 °C. We also analysed left-over blood samples from chimpanzees cared for at the Sanaga-Yong Rescue Centre (SYptt5, SYptt15, SYptt20, SYpte37, SYptt75, SYptt79 and SYptt82), which were obtained in the context of routine health examinations or for specific veterinary purposes. Samples were shipped in compliance with Convention on International Trade in Endangered Species of Wild Fauna and Flora regulations and country-specific import and export permits. DNA was extracted from faecal and blood samples using the QIAamp Stool DNA Mini Kit and QIAamp Blood DNA Mini Kit (Qiagen, Valencia, CA), respectively, described in detail in ref. 47.

### *Plasmodium* species identification

The *Plasmodium* species composition in ape faecal and blood samples was determined by SGS and phylogenetic analysis^1^,^47^. Briefly, faecal and blood DNA was end point diluted in 96-well plates, and the dilution that yielded <30% wells with positive PCR reactions was used to generate between 2 and 174 different SGS sequences per sample (according to a Poisson distribution, the DNA dilution that yields PCR products in <30% of wells contains one amplifiable template per positive PCR >83% of the time). Amplification products were gel purified, and sequenced directly without interim cloning. Sequences that contained double peaks as an indicator of more than one amplified template were discarded. Different genomic loci were amplified, including portions of mitochondrial (cytochrome B), nuclear (erythrocyte binding antigens *eba165* and *eba175*, 6-cysteine protein *p47* and *p48/45*, lactate dehydrogenase, reticulocyte-binding protein homolog 5) and apicoplast (caseinolytic protease C) genes. All relevant primers are provided in Supplementary Table 4. For each genomic region, up to 73 single template-derived amplicons were sequenced and their species origin was identified by phylogenetic analysis (Supplementary Table 1). This analysis identified seven blood samples and one faecal sample to represent single-species infections of *P. reichenowi* (SYptt15, 46 SGS sequences), *P. gaboni* (SYptt5, 86 SGS sequences; SYpte37, 59 SGS sequences; SYptt75, 122 SGS sequences; SYptt82, 59 SGS sequences), *P. billcollinsi* (SYptt20, 174 SGS sequences; SYptt79, 16 SGS sequences) and *P. praefalciparum* (GTggg140; 2 SGS sequences), although many of these specimens contained multiple variants (haplotypes) of the respective species. Three other faecal samples (GTggg118, KApts1680 and DGptt540) contained more than one ape *Laverania* species, and one included an additional non-*Laverania* species of unknown origin (Supplementary Table 1).

### PCR amplification of *var* genes

DBL domains were amplified, cloned and sequenced (see, for example, refs 30, 31) using conventional (rather than limiting dilution) PCR. Different primers sets, listed below, were used to amplify 2.5 μl of faecal or blood derived DNA in a 25-μl reaction volume, containing 0.5 μl dNTPs (10 mM of each dNTP), 10 pmol of each primer, 2.5 μl PCR buffer, 0.1 μl BSA solution (50 mg ml^−1^) and 0.25 μl expand long template enzyme mix (Expand Long Template PCR System, Roche). Most samples were subjected to single-round amplification with previously published primers, including DBLα-5′ (5′-GCACGAAGTTTTGCAGATATWGG-3′) and DBLα-3′ (5′-AARTCTTCKGCCCATTCCTCGAACCA-3′)^31^, or DBLαAF′ (5′-GCACGMAGTTTYGC-3′) and DBLαBR (5′-GCCCATTCSTCGAACCA-3′)^30^. Only three samples were amplified with additional primers, including C1DBLαAF′ (5′-GCACGVAGTTTTGC-3′) and C1DBLαBR (5′-GCCCATTCSTSGAACCA-3′), and C2DBLAF (5′-AARTAHAGTTTTGCTGATTTARG-3′) and C2DBLAR (5′-TTCGGACCATTCGKCWAWCCA-3′), respectively, or by nested PCR. The C2DBLAF and C2DBLAR primers were designed to specifically amplify *P. gaboni* DBL tags using an alignment of select whole-genome amplification derived contigs of SYpte37. Cycling conditions included an initial denaturing step of 2 min at 94 °C, followed by 35–60 cycles of denaturation (94 °C, 10 s), annealing (50–55 °C, 30 s) and elongation (68 °C, 1 min), followed by a final elongation step of 10 min at 68 °C. Both single-round and nested PCR-derived amplicons were gel purified and subcloned into pGEM-T Easy (Promega) or PCR4 TOPO (Life Technologies) plasmid vectors. Positive clones were sequenced, and analysed using SEQUENCHER (Gene Codes Corporation, Ann Arbor, MI) or Lasergene (DNASTAR) software.

### Criteria of *var* gene sequence selection

Amplified *var* DBL sequences were inspected for primer sequences (which were removed from final sequences) and the presence of a single intact ORF; sequences lacking an intact ORF or identifiable 5′ and 3′ primer sequences were discarded. To remove *Taq* polymerase errors in cloned DBLα *var* tag sequences, a neighbour-joining tree was constructed for each sample and sequences differing by less than three nucleotides were condensed into a single-consensus sequence. Thus, for each sample only unique DBLα var tag haplotypes were analysed.

### Network analysis

A short region of *var* gene sequence within the DBLα domain, which we refer to as a ‘tag,' comprises three conserved HBs (HB3, HB5 and HB2) separated by two HVRs^19^. We identified HVRs using a sequence entropy approach, modifying a previously published procedure^21^ to accommodate ape *Laverania* sequences. To extract highly variable sequence content for further study, we identified and removed the three conserved HBs from the 3′-end, middle and 5′-end of each tag sequence. This was carried out by first aligning all sequences first to HB3 without inserting any gaps mid-sequence (step 1), that is, we required that all sequences align at and only at HB3. Next, we calculated the Shannon entropy of the aligned sequences at each position (step 2) and scanned from HB3 towards the centre of the tag to find the first position *p* at which entropy was >2 bits (step 3) such that each subsequent position also had entropy >2 bits. Finally, we retained all sequences from *p* towards the centre of the tag (step 4). Steps 1–4 were repeated for HB2, thus removing low-entropy HBs from the ends of each sequence. Second, we removed conserved central sequence content, splitting the tag into two HVRs. We repeated steps 1 and 2 with HB5. We then scanned from HB5 towards each end of the tag, finding the first position *p* in each direction with entropy >2 bits such that each subsequent position had entropy >2 bits, and retained everything from *p* towards the end of the tag. All steps are shown graphically in Supplementary Fig. 7. The high-entropy HVR between HB3 and HB5 is referred to as the left HVR and the high-entropy HVR between HB5 and HB2 is referred to as the right HVR.

Two types of networks were created. First, networks of *var* sequences were generated by assigning each HVR sequence to a node and placing a link between two nodes when their corresponding sequences shared a block of length *L* or greater at the amino-acid level. *L=7* for left HVR and *L=6* for right HVR, based on null model calculations^21^. Figures were produced using force-directed layouts in *webweb* software v3.1 (http://danlarremore.com/webweb). Second, bipartite networks of both *var* genes and their shared blocks were created by assigning each HVR sequence and each shared block of length *L* or greater to a node, and placing a link between a sequence node and a shared block node if the block is present in the sequence. These bipartite networks are related to the other type of network via one-mode projection. Community detection was performed using the biSBM method applied to bipartite networks of sequences and their shared amino-acid substrings^48^.

### *k*-mer stackup analysis

Within an amino-acid sequence, we refer to any contiguous substring of length *k* amino acids as a *k*-mer. All *k*-mers were extracted from all sequences, noting the starting position (normalized to the total length of the sequence). For Supplementary Fig. 2a, all *k*-mers from *P. falciparum* and *P. reichenowi* were sorted by their frequency of appearance, and stacked histograms of their starting positions were created with 50 bins. For Supplementary Fig. 2b, all *k*-mers from each of *P. falciparum, P. reichenowi, P. gaboni* and *P. billcollinsi* were sorted by their presence across species, and stacked histograms of their starting positions (relative to the species indicated at the top of each plot) were created with 50 bins.

### Bayesian *k*-mer analysis

A window of length *k* was scanned across each amino-acid sequence from *P. falciparum* and *P. reichenowi* monoinfections, extracting all length *k* substrings. Some substrings appeared in sequences from both species, while others were species specific. This analysis, derived and developed in detail below, estimates the overlap in populations of tag sequences using Bayesian statistics to correctly extrapolate the parameters of the conjugate prior distribution that characterizes the overlap from limited sample data^49^.

For this analysis, we examine 296 DBLα tag sequences from *P. falciparum* and 94 from *P. reichenowi*. Each sequence is a string of amino acids, so from a sequences of length *N*, we can extract *N−k+1* substrings (that is, *k*-mers, or words) of length *k*. In what follows, we use *k*=7 for all examples. (Other values of *k* may be used, and results do not depend sensitively on moderate *k*; we tested *k*∈[5, 15].) The 390 total sequences comprise 45,731 words for *k*=7, but some words appear in multiple sequences; the total number of unique words is 22,431. This indicates that, on average, each word appears approximately two times across all 390 sequences. In fact, the distribution is highly heterogeneous: 70% of words appear only once, 16% appear only twice and 6% appear only three times, meaning that 92% of words appear in only 1–3 of the total 390 sequences. This heterogeneity, depicted in Supplementary Fig. 8, makes it difficult to decide whether these two sets of sequences are drawn from distinct populations.

Some words are shared by both *P. falciparum* and *P. reichenowi* (8%), some are unique to *P. falciparum* (65%) and the rest unique to *P. reichenowi* (27%). If only 8% of (length 7) words are shared by both species, one might conclude that the populations of words are well separated. However, owing to the massive diversity of words in both species, this interpretation is incorrect. Instead of calculating the overlap between species for our data set, we wish to estimate the overlap for the global populations of *P. falciparum* and *P. reichenowi*.

Before the mathematical formulation, we advance the following helpful analogy, by imagining each word as a biased coin. Suppose we have a large bag of coins and each coin has a biased probability of landing on heads. Further, imagine that the biases are not all the same, but are instead drawn from some distribution. We wish to estimate the distribution, so we take the coins, one by one, and flip them, writing down which coin was flipped and whether it lands on heads or tails each time. However, for 70% of the coins, we only get one flip. For 16% of the coins, we only get two flips, and for 6% of the coins we only get three flips and so on. When estimating the distribution of *p*, we must take into account the number of flips observed for each coin.

Given our small sample from the distribution, we wish to approximate the global distribution of values of *p*_*i*_. This will tell us how much the populations overlap. Our data consist of *f*_*i*_ and *r*_*i*_, the numbers of observations of word *i* in *P.* f*alciparum* and *P.* r*eichenowi*, respectively. We model the assignment of each occurrence of word *i* to *P. reichenowi* as an independent Bernoulli trial, with parameter *p*_*i*_. Let the set of *p*_*i*_ be Beta distributed with parameters *α* and *β*, where we use the Beta distribution because it is the conjugate prior of the Bernoulli distribution. Then, the likelihood of observing data {*x*_*i*_}, given the parameters, is


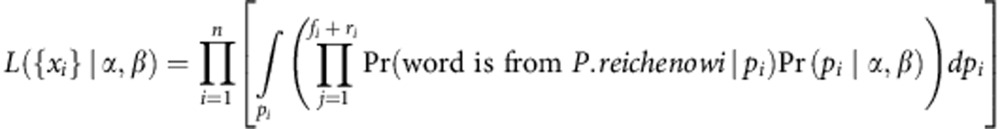


which may be integrated using beta functions *B* to get


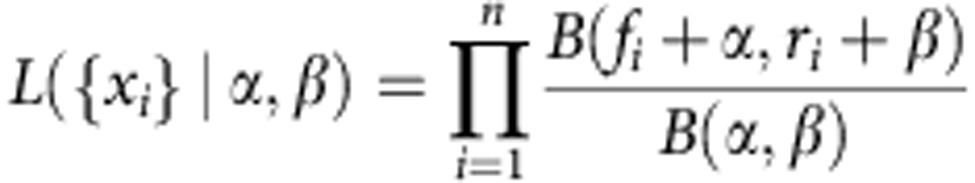


Taking a log yields


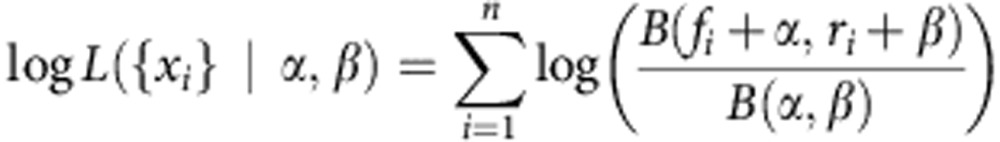


This log-likelihood function is related to a solution to an analogous problem from the domain of probabilistic competition dynamics^49^ in which two teams were competing for points over the course of many competitions. We maximize it in MATLAB using the fminsearch function, using the observed *f*_*i*_ and *r*_*i*_ values.

### Pairwise distance analysis

Protein sequences were aligned pairwise using MUSCLE v3.8.1 (ref. 50), and Hamming distances (number of sites at which the two aligned sequences differ) were calculated neglecting gaps at both ends of the alignment to adjust for variable sequence lengths. Hamming distances were alternatively calculated by counting a contiguous block of gaps as a single difference, with no qualitative difference in results.

### Blocksharing

We quantified sequence conservation from one particular sequence to an ensemble of others by scanning a window of length *k* across the particular sequence and computing the fraction of sequences in the ensemble containing each *k*-mer or block. This produces a measure of conservation between 0 and 1 in the frame of reference of the particular sequence; Fig. 1b shows this blocksharing for the DBLα domain of *DD2var11* compared with the background of data in ref. 19; *k*=7.

### CP group analysis

*Var* tag sequences can be classified according to the number of cysteine residues as well as sequence content at defined ‘positions of limited variability (PoLV)'^30^. In the *var* sequence literature, these are referred to as Cys-PoLV groups, or simply CP groups. We identified CP groups with a MATLAB script according to the following definitions: group 1: MFK* at PoLV, two cysteine residues; group 2: *REY at PoLV2, two cysteine residues; group 3: two cysteine residues, not group 1 or 2; group 4: four cysteine residues, not group 5; group 5: *REY at PoLV2, four cysteine residues; group 6: one, three, five, or six cysteine residues. Histograms of cysteine counts and CP groups are shown in Supplementary Fig. 6.

### *P. gaboni* select whole-genome amplification

DNA was extracted from two chimpanzee blood samples (SYpte37 and SYptt75) identified as *P. gaboni* single-species infections by single-genome sequencing (Supplementary Table 1) and subjected to select whole *Plasmodium* genome amplification as described^27^,^28^. Briefly, total DNA (100 ng–1 μg) was digested using the methylation dependent restriction enzymes MspJI and FspEI in multiple replicates. The digestion products were amplified using phi29 polymerase and one of two primer sets consisting of 10 primers (8–12 nt in length each) designed to bind frequently and broadly to the *P. falciparum* genome but only rarely to the chimpanzee genome^28^. A amount of 50 ng of first round product was reamplified in a second reaction using the second primer set. Replicates were pooled and a short insert library was constructed using the TruSeq DNA PCR-Free Sample Preparation Kit (Illumina) and sequenced using a MiSeq Reagent Kit V2 (500 cycles; Illumina) to generate 250 bp paired end reads. Reads were mapped to the *P. falciparum* 3D7 reference genome using Geneious (Biomatters Limited, Auckland, New Zealand), and subjected to guided assembly using Velvet Columbus^51^. For SYptt75, contigs produced by Velvet were aligned to the reference and the resulting core *P. gaboni* draft genome was iteratively corrected manually and using PAGIT v1.0 (ref. 52). All reads from SYptt75 and SYpte37 were mapped to this draft reference and reads that could not be mapped were assembled separately using Spades v3.1.1 (ref. 53).

### Putative *var* gene identification *var* domain analysis

Due to the hypervariability of *var* sequences, *P. gaboni* reads did not map to *var* gene containing regions of the *P. falciparum* 3D7 reference genome, nor were *var* genes readily identified in the SYptt75 core *P. gaboni* genome. A search for contigs containing *var*-like sequence was therefore performed on unplaced SYptt75 and SYpte37 contigs (produced by either Velvet or Spades in a reference-independent manner). Specifically, tblastn and tblastx searches were performed using all *P. gaboni* unplaced contigs against a database of available full-length *P. falciparum* 3D7 and *P. reichenowi* CDC1 *var* genes. Genes and ORFs were identified in the top hits manually and by Augustus v2.5.5 gene prediction^54^, and pblast searches using the resulting amino-acid sequences were again performed against the translated *P. falciparum* and *P. reichenowi var* gene database. Hits were then submitted to the VarDom 1.0 server (http://www.cbs.dtu.dk/services/VarDom/)^19^ for domain identification and classification.

The *P. gaboni* ortholog of PF3D7_0113800 was identified in the draft SY75 sequence by blast homology to PF3D7_0113800. Gene annotation was performed using RATT^55^ with manual correction.

### Neighbour-joining tree construction

Protein sequence tags were aligned using MUSCLE v3.8.1 (ref. 50) and the phylogeny were created using the neighbour-joining distance method, with Poisson distances, as implemented in Seaview 4.4.0 (ref. 56).

### DBLx identification and classification

DBLx domains were identified as those tags that (i) were <90 residues in length, and (ii) began with residues NI, DF or DM. Those that began with residues NI were further classified as DBLx1 and those that began with DF or DM as DBLx2. A total of 100% of DBLx sequences also featured a lysine residue (K) in the fourth position of the tag instead of the DBLα arginine (R). Sequence logos are shown in Supplementary Fig. 3.

## Additional information

**Accession codes:** DBL *var* tag sequences have been deposited in the GenBank nucleotide database under accession codes KP167140 to KP167147, and KJ801976 to KJ802011. *P. gaboni* unplaced contigs with DBL domains have been deposited in the GenBank nucleotide database under accession codes KP879220 to KP879255. *P. gaboni* unplaced contigs with ATS domains have been deposited in the GenBank nucleotide database under accession codes KT343259 to KT343272.

**How to cite this article:** Larremore, D. B. *et al*. Ape parasite origins of human malaria virulence genes. *Nat. Commun.* 6:8368 doi: 10.1038/ncomms9368 (2015).

## Supplementary Material

Supplementary Information Supplementary Figures 1-8, Supplementary Tables 1-4 and Supplementary References

## Figures and Tables

**Table 1:** *Var* gene-like structures in *P. gaboni* whole-genome contigs.

Sample	Total *var*-like DBLs identified	Number of DBLs (number of contigs)					DBL classification*			*var*-like ATS
		**1-DBL**	**2-DBL**	**3-DBL**	**4-DBL**	**5-DBL**	**DBLɛ**	**DBLζ**	**unclassified**	
SYpte37	15	8 (8)	4 (2)	3 (1)	—	—	2	—	13	0
SYptt75	40	23 (23†)	8 (4)	—	4 (1)	5 (1)	12	2	26	16‡

ATS, acidic terminal segment

^*^DBLα–δ domains according to the classification by Rask *et al*.^19^ were not identified. In addition, we found no evidence of DBLα-CIDR domain pairs.

^†^Includes the three-exon single-DBL containing contig shown in Fig. 6.

^‡^Includes the three contigs with *var*-like DBL-TM̂ATS multi-domain (two exons) structure
