# Supplementary material for: Ape parasite origins of human malaria virulence genes
Source: Nat Commun. 2015 Oct 12;6:8368. doi: 10.1038/ncomms9368 (PMC4633637; doi:10.1038/ncomms9368)
Supplement: Supplementary Information — Supplementary Figures 1-8, Supplementary Tables 1-4 and Supplementary References [file ncomms9368-s1.pdf]

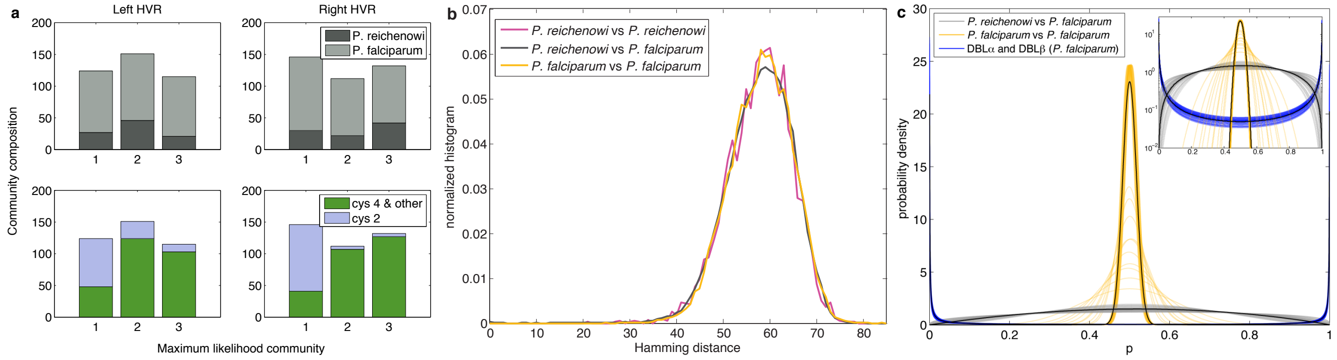

Supplementary Figure 1: Additional analyses of *P. falciparum*-*P. reichenowi var* tag sequences. DBLα tags of *P. falciparum* and *P. reichenowi var* genes are indistinguishable using network and pair-wise distance analyses, and highly overlapping using a Bayesian *k*-mer analysis. **(a)** Bars show the composition of *P. falciparum*-*P. reichenowi* network (Fig. 2) community structures by species (top row) and by cysteine count classification (bottom row). **(b)** Normalized histograms of pair-wise Hamming distances (number of aligned positions at which two sequences differ) between *var* tags are shown, with colors corresponding to the *Plasmodium* species compared as indicated. **(c)** Probability density of posterior distributions of *k*-mer sharing parameter *p* (Methods). Each set of lines shows 100 distributions using independently estimated maximum likelihood parameters; black lines indicate distributions using corresponding averages of maximum likelihood parameters. Inset shows identical data on a log axis. Distributions peaked at *p*=0.5 indicate maximally overlapping *k*-mers; distributions peaked at 0 and 1 indicate minimally overlapping *k*-mers. Representative plot with *k*=7 shown.

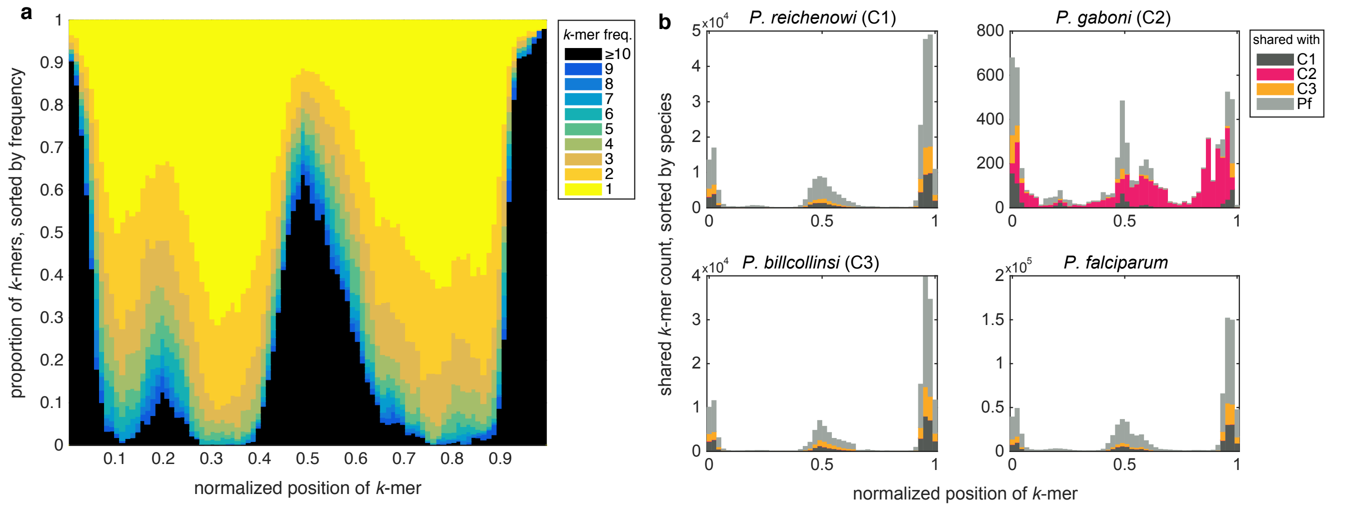

Supplementary Figure 2: *k*-mer frequency and *k*-mer inter-species sharing in *Laverania var* gene sequences. Data shown represent all sequences from all *Laverania* single-species samples with *k*=7. Horizontal axes correspond to starting position of a *k*-mer and vertical axes correspond to stacked proportions (subplot a) or absolute numbers (subplot b) of *k*-mers, sorted as follows. **(a)** Proportions of *k*-mers sorted and colored by observed frequency in all tag sequences. Observed frequency of one (bright yellow) indicates a unique *k*-mer; All frequencies greater than 10 are binned together as black; other colors as indicated in legend. **(b)** Counts of *k*-mers in each of four species, sorted and colored by aggregate counts in tags from other species.

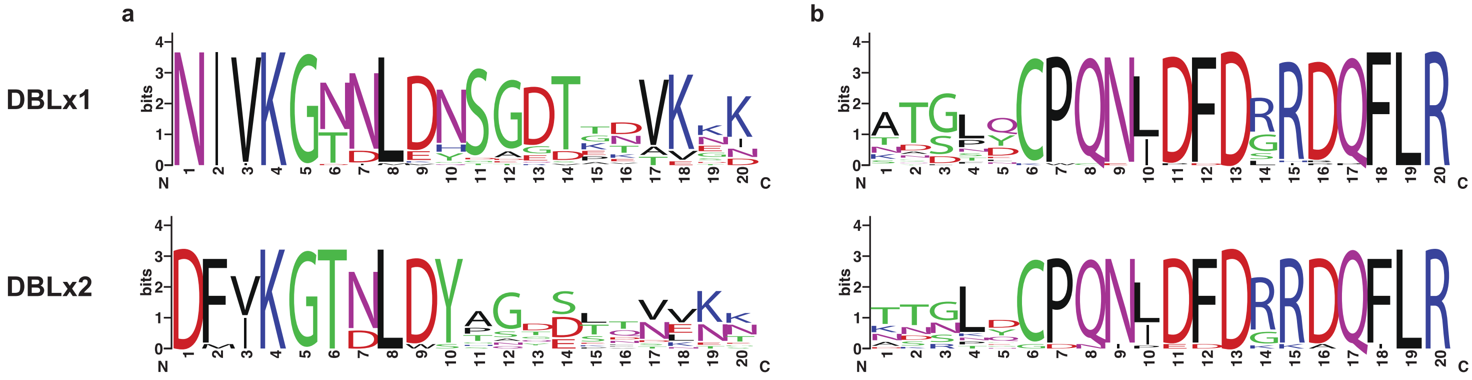

Supplementary Figure 3: Sequence motifs within conserved regions of DBLx tag sequences. DBLx tags may be divided into two potential sub-groups, based on differences in residues at the N-terminus. **(a)** The first 20 residues of the N-terminus of the tag sequences. **(b)** The last 20 residues of the C-terminus of the tag sequences.

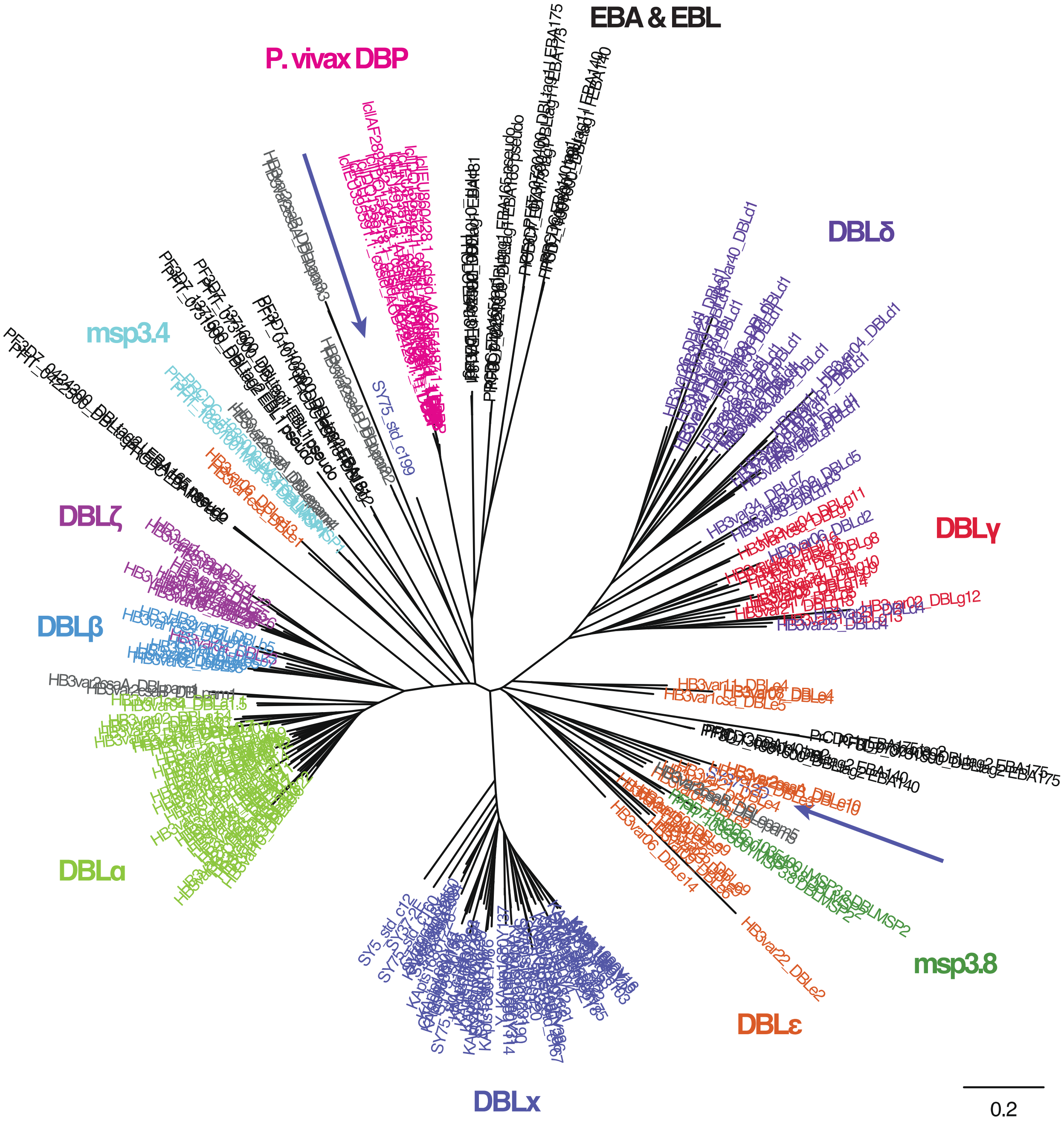

Supplementary Figure 4: Neighbor joining tree of DBLx tag sequences in the context of *var* and non-*var* DBL domain tags. *Var* DBL domain tags are from P. *falciparum* parasite HB3 and non-*var* DBL domains are from other parasites as indicated (see Supp. Table 3). Colors and labels correspond to domain classes. Grey unlabeled sequences are from DBLpam or DBLεpam domains. Arrows indicate DBLx sequences that fall far from others. This tree summarizes patterns of protein similarity and is not a depiction of evolutionary history.

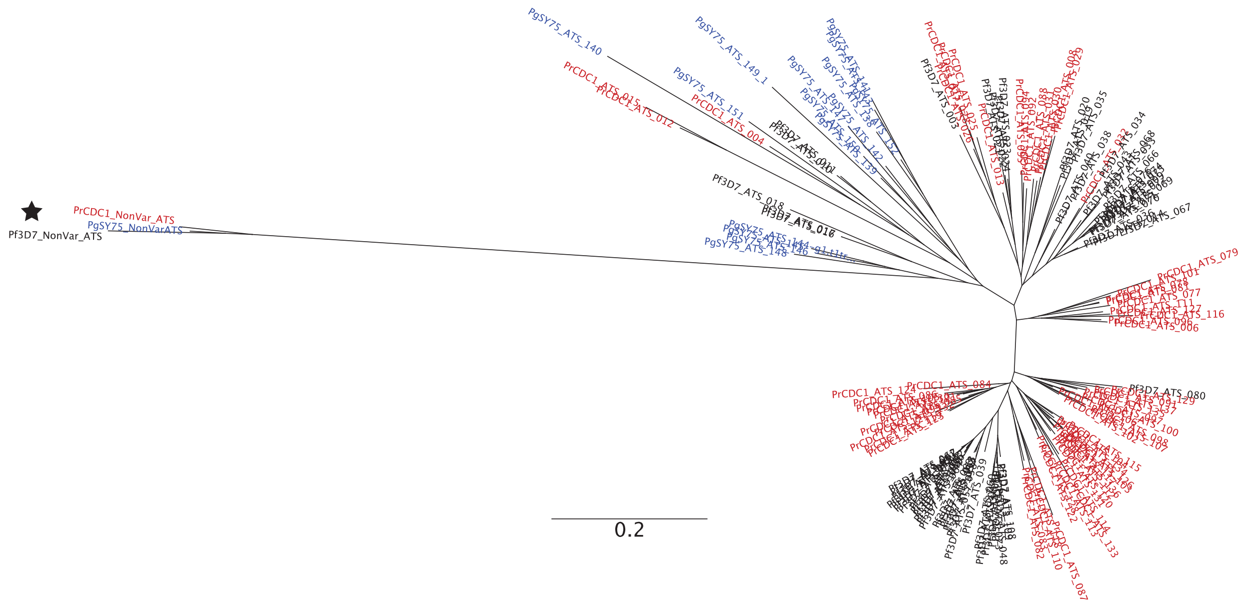

Supplementary Figure 5: Neighbor joining tree of ATS and ATS-like amino acid sequences. ATS domains are from *P. falciparum* parasite 3D7, *P. reichenowi* parasite PrCDC, *and P. gaboni* parasite SYptt75. Included are *var* and *var*-like genes as well as the *P. falciparum* PF3D7_0113800 gene and its *P. reichenowi* and *P gaboni* orthologues previously reported to contain ATS-like sequences[1]. Tip labels are colored by species with *P. falciparum, P. reichenowi*, and *P. gaboni* shown in black, red, and blue, respectively. The star denotes ATS-like sequences from *P. falciparum, P. reichenowi*, and *P. gaboni* orthologs of PF3D7_0113800. Due to ATS-like domain diversity, only the more conserved N-terminal region, corresponding to HB41 and HB43 of the *P. falciparum var*-ATS domain (see Figure 5), was used in the alignment. *Var* and *var*-like ATS sequences that did not span this entire region were excluded from the analysis. This tree summarizes patterns of protein similarity and is not a depiction of evolutionary history.

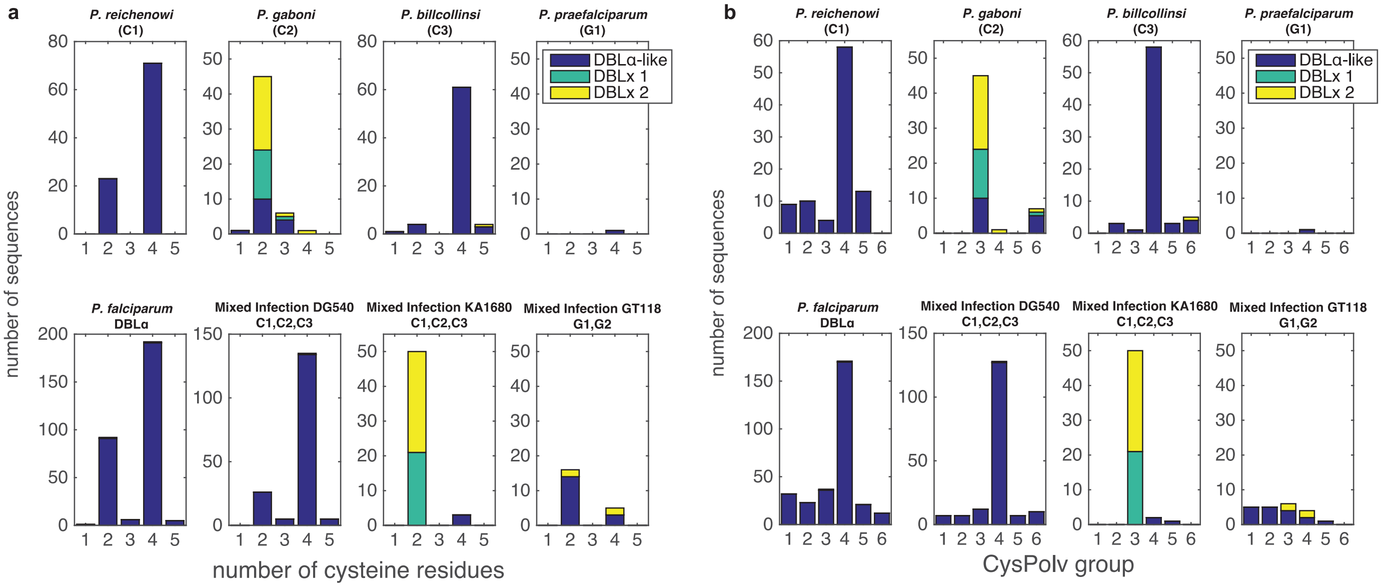

Supplementary Figure 6: Repertoire structure of *Laverania var* tag sequences by cysteine counts and CysPolv (CP) groups. Histograms show **(a)** the number of cysteine residues in each tag sequence, and **(b)** CP groups[2] for tag sequences, sorted by sample. Repertoires appear stably structured for *P. falciparum, P. reichenowi,* and *P. billcollinsi*. Data for other species and samples are too few to draw conclusions but are shown. DBLα-like, DBLx1, and DBLx2 tags are colored as indicated in legend.

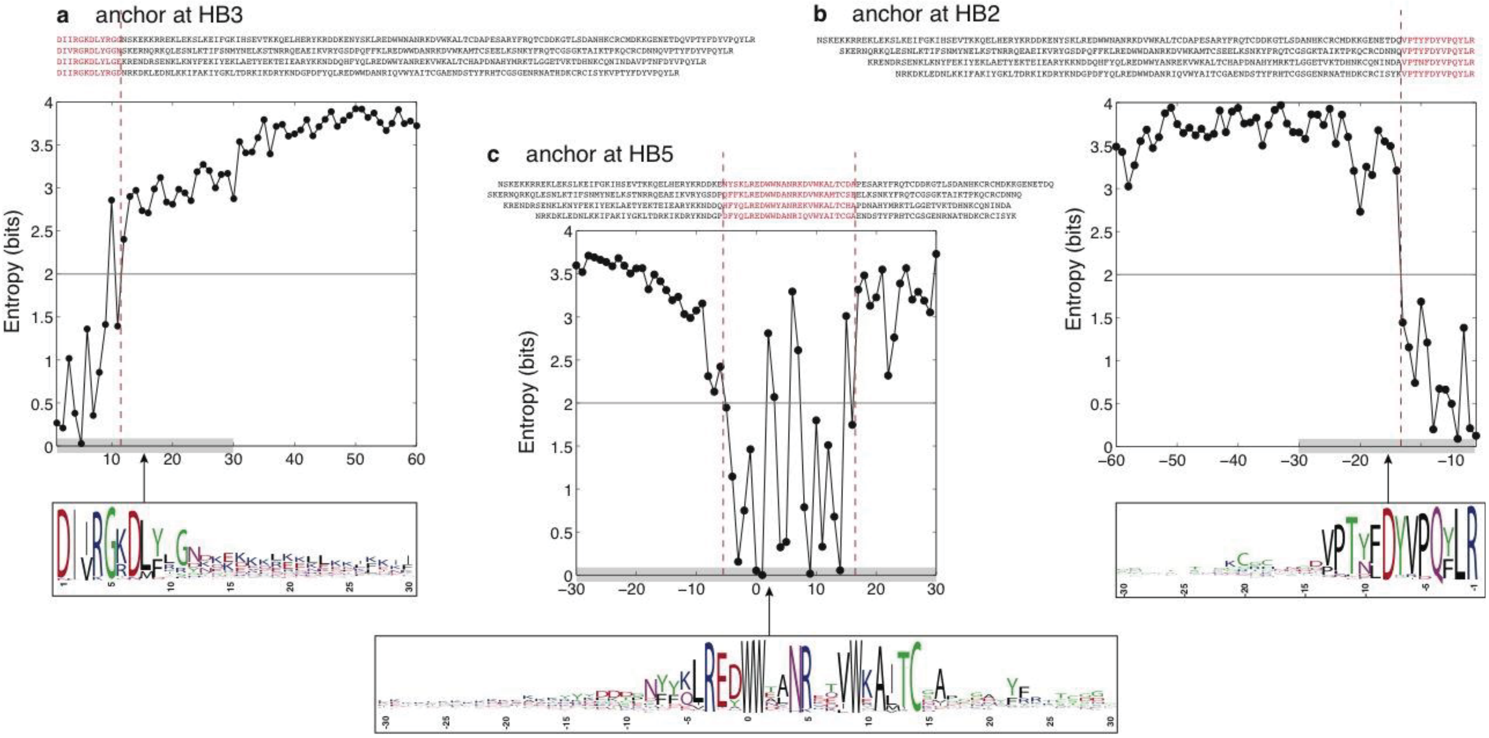

Supplementary Figure 7: Identification of Highly Variable Regions (HVRs) in DBL tag sequences. Left and Right HVRs were determined by "anchoring" or aligning sequences to a homology block (HB), calculating sequence entropy, and removing conserved sequence content (see Methods). This process is repeated for (a) HB3, (b) HB2, and (c) HB5. In each panel, four sample sequences are anchored to elucidate this process: black text indicates retained sequence content; red text indicates removed content. Red dashed line(s) corresponds to position(s) of cutoff, as identified from plot of sequence entropy. Sequence entropy plots were generated from all *Laverania* data. Corresponding sequence "logo" plots are shown for the 30 residues adjacent to each anchor point, corresponding to shaded regions in plots.

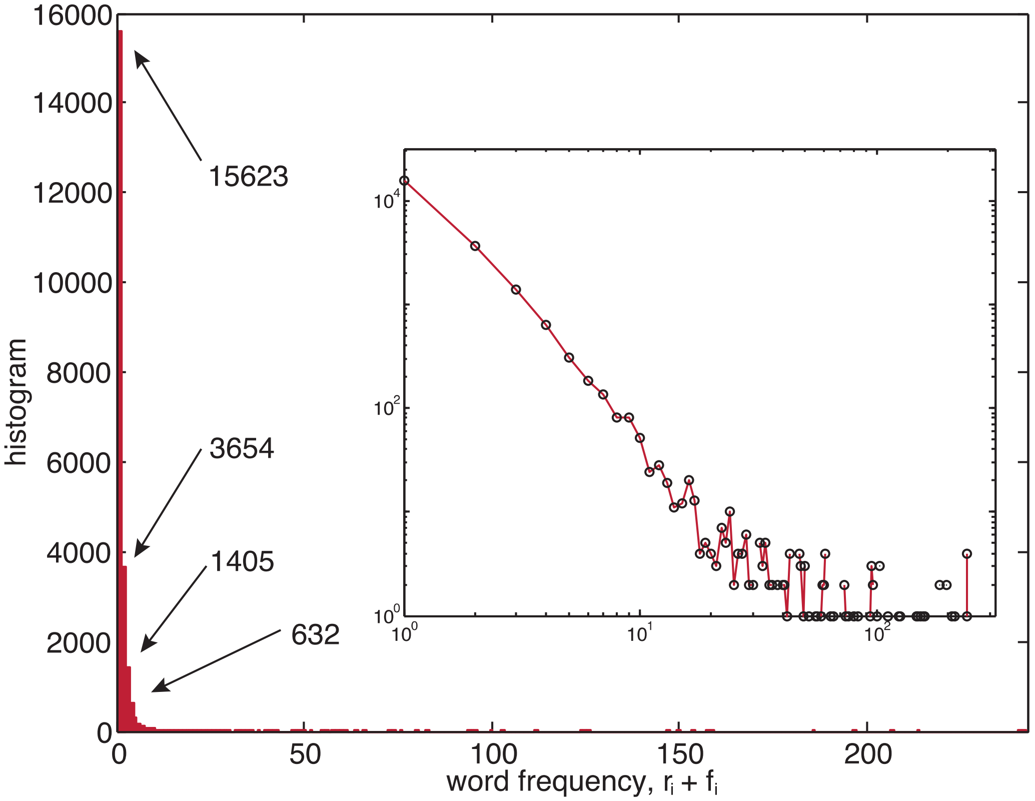

Supplementary Figure 8: **Histogram of word frequencies in *P. falciparum* and *P. reichenowi* sequences**. Word (or *k*-mer) frequencies, (calculated as described in Methods, $r_i + f_i$) shows that most words are unique, i.e. $r_i + f_i = 1$. Numerical labels with arrows indicate bar heights for the first four bars. Inset shows identical data on log-log axes to highlight the breadth of the distribution. While this plot shows $k = 7$, plots for other values of k show similarly broad distributions.

Supplementary Table 1. *Laverania* species composition in ape samples

Sample code[a]	Country[b]	Collection Date (mm/dd/yy)	Sample	*cytB*[c] SGS (hap)[d]	*cytB*[c] *species*[e]	*eba165*[c] SGS (hap)[d]	*eba165*[c] *species*[e]	*eba175*[c] SGS (hap)[d]	*eba175*[c] *species*[e]	*p47*[c] SGS (hap)[d]	*p47*[c] *species*[e]	*p48/45*[c] SGS (hap)[d]	*p48/45*[c] *species*[e]	*ldh*[c] SGS (hap)[d]	*ldh*[c] *species*[e]	*RH5*[c] SGS (hap)[d]	*RH5*[c] *species*[e]	*clpC*[c] SGS (hap)[d]	*clpC*[c] *species*[e]	Species composition[e] (# of total SGS amplicons)
SYptt15	Cameroon	7/11/12	Blood	11 (1)	C1 (11)	N/A	N/A	N/A	N/A	7 (1)	C1 (7)	N/A	N/A	12 (1)	C1 (12)	4 (1)	C1 (4)	12 (1)	C1 (12)	C1 (46)
SYptt5	Cameroon	5/7/11	Blood	5 (1)	C2 (5)	8 (1)	C2 (8)	5 (1)	C2 (5)	10 (2)	C2 (10)	8 (1)	C2 (8)	37 (2)	C2 (37)	2 (1)	C2 (2)	11 (1)	C2 (11)	C2 (86)
SYpte37	Cameroon	8/3/12	Blood	8 (1)	C2 (8)	3 (1)	C2 (3)	6 (1)	C2 (6)	5 (2)	C2 (5)	4 (1)	C2 (4)	27 (1)	C2 (27)	3 (1)	C2 (3)	3 (1)	C2 (3)	C2 (59)
SYptt75	Cameroon	10/17/13	Blood	15 (3)	C2 (15)	5 (1)	C2 (5)	3 (2)	C2 (3)	53 (4)	C2 (53)	8 (2)	C2 (8)	29 (4)	C2 (29)	N/A	N/A	9 (1)	C2 (9)	C2 (122)
SYptt82	Cameroon	10/28/13	Blood	12 (1)	C2 (12)	8 (1)	C2 (8)	8 (1)	C2 (8)	7 (1)	C2 (7)	2 (1)	C2 (2)	20 (1)	C2 (20)	N/A	N/A	2 (1)	C2 (2)	C2 (59)
SYptt20	Cameroon	2/2/12	Blood	4 (1)	C3 (4)	42 (2)	C3 (42)	6 (1)	C3 (6)	7 (1)	C3 (7)	8 (1)	C3 (8)	23 (1)	C3 (23)	73 (2)	C3 (73)	11 (1)	C3 (11)	C3 (174)
SYptt79	Cameroon	8/27/13	Blood	1 (1)	C3 (1)	6 (2)	C3 (6)	4 (1)	C3 (4)	1 (1)	C3 (1)	2 (1)	C3 (2)	2 (2)	C3 (2)	N/A	N/A	N/A	N/A	C3 (16)
GTggg140	RC	4/12/08	Faeces	2 (2)	G1 (2)	N/A	N/A	N/A	N/A	N/A	N/A	N/A	N/A	N/A	N/A	N/A	N/A	N/A	N/A	G1 (2)
GTggg118	RC	10/24/03	Faeces	42 (6)	G1 (40), G2 (2)	1 (1)	G1 (1)	7 (3)	G1 (5), G2 (2)	2 (1)	G1 (2)	1 (1)	G1 (1)	2 (2)	G1 (2)	1 (1)	G1 (1)	1 (1)	G1 (1)	G1 (53), G2 (4)
DGptt540	Cameroon	9/3/04	Faeces	21 (9)	C1 (12), C3 (8), Unk (1)	6 (3)	C1 (6)	14 (5)	C1 (10), C3 (4)	10 (4)	C1 (10)	4 (3)	C1 (3), C3 (1)	2 (2)	C1 (2)	9 (4)	C1 (9)	8 (6)	C2 (5), C3 (2), Unk (1)	C1 (52), C2 (5), C3(15), Unk (2)
KApts1680	DRC	3/9/07	Faeces	32 (8)	C1 (1), C2 (31)	17 (5)	C2 (17)	10 (7)	C1 (2), C2 (6), C3 (2)	34 (5)	C2 (34)	3 (2)	C2 (3)	6 (4)	C2 (6)	9 (1)	C2 (9)	3 (3)	C2 (2), C3 (1)	C1 (3), C2 (108), C3 (3)

[a]Blood samples were collected opportunistically from individually known chimpanzees housed at a Wildlife Rescue Center (SY). Fecal samples were collected from wild-living apes, with their species and subspecies origin determined by mitochondrial DNA analysis (*ptt, P. t. troglodytes; pte, P. t. ellioti; pts, P. t. schweinfurthii; ggg, G. g. gorilla*); a two-letter code indicates the sample's field site of origin[3].

[b]RC, Republic of the Congo; DRC, Democratic Republic of the Congo.

[c]PCR amplified loci in the *Plasmodium* mitochondrial (cytB), nuclear (*eba165, eba175, p47, p48/45, ldh,* RH5) and apicoplast (*clpC*) genomes: *cytB*, cytochrome B; *eba165* and *eba175*, erythrocyte binding antigens EBA165 and EBA175; *p47* and *p48/45*, *Plasmodium* 6-cysteine proteins P47 and P48/45; *ldh*, lactase dehydrogenase; RH5, reticulocyte-binding protein homolog 5; *clpC*, caseinolytic protease C.

[d]SGS, number of single genome derived sequences; brackets indicate the number of distinguishable haplotypes (hap). N/A, not available.

[e]Ape *Laverania* species present in sample: C1, *P. reichenowi*; C2, *P. gaboni*; C3, *P. billcollinsi*; G1, *P. praefalciparum*; G2, *P. alderi*; G3, *P. blacklocki*; Unk, unknown non-*Laverania* species. Brackets indicate the number of independent SGS derived sequences for that ape *Laverania* species.

Supplementary Table 2. Amplification of *var* DBL domains from ape samples

Sample code[a]	Species composition[b]	*var* DBL haplotypes[c]	First round primers	Second round primers	GenBank Accession No.
SYptt15	C1	7	upsA750/BetaR	DBLαAF'/DBLαBR	KJ801976-KJ801982
		30	C1DBLαAF/C1DBLαBR	N/A	KJ801983-KJ802011
SYptt5	C2	5	DBLα-5'/DBLα-3'	N/A	KP167365-KP167369
SYpte37	C2	15	C2DBLAF/C2DBLAR	N/A	KP167410-KP167424
SYptt75	C2	15	DBLα-5'/DBLα-3'	N/A	KP167425-KP167439
SYptt82	C2	1	DBLα-5'/DBLα-3'	N/A	KP167471
		1	C2DBLAF/C2DBLAR	NA	KP167470
SYptt20	C3	31	DBLα-5'/DBLα-3'	N/A	KP167370-KP167409
		9	C2DBLAF/C2DBLAR	N/A	
SYptt79	C3	30	DBLα-5'/DBLα-3'	N/A	KP167440-KP167469
GTggg140	G1	1	DBLα-5'/DBLα-3'	N/A	KP167332
GTggg118	G1, G2	21	DBLα-5'/DBLα-3'	N/A	KP167311-KP167331
DGptt540	C1-3, Unk	162	DBLα-5'/DBLα-3'	N/A	KP167140-KP167310
		2	upsA750/BetaR	DBLαAF'/DBLαBR	
		3	upsB/BetaR	DBLαAF'/DBLαBR	
		1	upsA750/DBLαBR	DBLαAF'/PTYF3	
		3	upsB/DBLαBR	DBLαAF'/PTYF3	
KApts1680	C1-3	32	DBLα-5'/DBLα-3'	N/A	KP167333-KP167364

[a]Blood samples were collected opportunistically from individually known chimpanzees housed at a Wildlife Rescue Center (SY). Fecal samples were collected from wild-living apes, with their species and subspecies origin determined by mitochondrial DNA analysis (*ptt, P. t. troglodytes; pte, P. t. ellioti; pts, P. t. schweinfurthii; ggg, G. g. gorilla*); a two-letter code indicates the sample's field site of origin[3].

[b]Ape *Laverania* species composition as determined in Supplementary Table 1: C1, *P. reichenowi*; C2, *P. gaboni*; C3, *P. billcollinsi*; G1, *P. praefalciparum*; G2, *P. alderi*; G3, *P. blacklocki*; Unk, unknown non-*Laverania* species.

[c]*var* DBL domains were amplified using single or nested PCR, cloned into a plasmid vector (pGEM-T Easy), sequenced, and grouped into different haplotypes using phylogentic analysis (Methods). For each sample, only unique haplotypes were analyzed.

Supplementary Table 3. List of non-*var* DBL domains included in Figure 3.

<table>
<tr><th>Group</th><th>Isolate</th><th>Header</th><th>Sequence</th></tr>
<tr><td rowspan="10">P. vivax DBP</td><td>DQ156518</td><td>>lcl|DQ156518.1_cdsid_AAZ81531.1_1 [protein=duffy binding protein]</td><td>LGDFGDIIMGTDMEGIGYSKVVENNLRSIFGTGKNAQQHRKQWWNESKAQIWTAMMYSVKKRLKGKFIWICKINVAVNIEPQIYR</td></tr>
<tr><td>AF289482</td><td>>lcl|AF289482.1_cdsid_AAG30849.1_1 [protein=duffy binding protein]</td><td>LGDFGDIIMGTDMEGIGYSKVVENNGRSIFGTGEKAQQRRKQRWNESKAQIWTAMMYSVKKRLKGNFIWICKINVAVNIEPQTYR</td></tr>
<tr><td>DQ156512 (Sal I)</td><td>>lcl|DQ156512.1_cdsid_AAZ81525.1_1 [protein=duffy binding protein]</td><td>LGDFGDIIMGTDMEGIGYSKVVENNLRSIFGTDEKAQQRRKQWWNESKAQIWTAMMYSVKKRLKGNFIWICKLNVAVNIEPQIYR</td></tr>
<tr><td>DQ156513</td><td>>lcl|DQ156513.1_cdsid_AAZ81526.1_1 [protein=duffy binding protein]</td><td>LGDFGDIIMGTDMEGIGYSKVVENNLRSIFGTGEKAQQHRKQWWNESKAQIWTAMMYSVKKRLKGNFIWICKLNVAVNIEPQIYR</td></tr>
<tr><td>EU395591</td><td>>lcl|EU395591.1_cdsid_ACB42429.1_1 [gene=DBP] [protein=duffy binding protein]</td><td>LGDFGDIIMGTDMEGIGYSEVVENNLRSIFGTGEQAQQRRKQWWNESKAQIWTAMMYSVKKRLKGNFIWICKINVAVNIEPQIYR</td></tr>
<tr><td>EU812894</td><td>>lcl|EU812894.1_cdsid_ACJ01724.1_1 [gene=dbp] [protein=Duffy binding protein]</td><td>LGDFGDIIMGTDMEGIGYSKVVENNLRSIFGTDEKAQQRRKQWWNESKAQIWTAMMYSVKKRLKGNFIWICKLNVAVNIEPQIYR</td></tr>
<tr><td>EU860428</td><td>>lcl|EU860428.1_cdsid_ACJ54187.1_1 [protein=duffy binding surface protein region II]</td><td>LGDFGDIIMGTDMEGIGYSKVVENNLRSIFGTGKNAQQHRKQWWNETKAQIWRAMMYSVKKRLKGNFIWICKINVAVNIEPQIYR</td></tr>
<tr><td>FJ491215</td><td>>lcl|FJ491215.1_cdsid_ACN69945.1_1 [gene=DBP-II] [protein=duffy binding surface protein region II]</td><td>LGDFGDIIMGTDMEGIGYSKVVENNLRSIFGTDEKAQQRRKQWWNESKAQIWTAMMYSVKKRLKGNFIWICKLNVAVNIEPQIYR</td></tr>
<tr><td>GU143917</td><td>>lcl|GU143917.1_cdsid_ACY91987.1_1 [protein=Duffy binding protein]</td><td>LGDFGDIIMGTDMEGIGYSEVVENNLRSIFGTGKNAQQHRKQWWNESKAQIWTAMMYSVKKRLKGKFIWICKINVAVNIEPQIYR</td></tr>
<tr><td>JN255576</td><td>>lcl|JN255576.1_cdsid_AFD18594.1_1 [protein=duffy-binding protein]</td><td>LGDFGDIIMGTDMEGIGYSKVVENNLRSIFGTDEKAQQRRKQWWNESKAQIWTAMMYSVKKRLKGNFIWICKLNVAVNIEPQIYR</td></tr>
<tr><td rowspan="6">EBA-175</td><td rowspan="2">3D7</td><td>>PF3D7_0731500_DBLtag1 | Plasmodium falciparum 3D7 | erythrocyte binding antigen-175 (EBA175) | protein | length=1502</td><td>FLDYGHLAMGNDMDFGGYSTKAENKIQEVFKGAHGEISEHKIKNFRKKWWNEFREKLWEAMLSEHKNNINNCKNIPQEELQITQ</td></tr>
<tr><td>>PF3D7_0731500_DBLtag2 | Plasmodium falciparum 3D7 | erythrocyte binding antigen-175 (EBA175) | protein | length=1502</td><td>FADIRDIIGGTDYWNDLSNRKLVGKINTNSNYVHRNKQNDKLFRDEWWKVIKKDVWNVISWVFKDKTVCKEDDIENIPQFFR</td></tr>
<tr><td rowspan="2">IT</td><td>>PFIT_0730400_DBLtag1 | Plasmodium falciparum IT | erythrocyte binding antigen-175 | protein | length=1502</td><td>FLDYGHLAMGNDMDFGGYSTKAENKIQEVFKGAHGEISEHKIKNFRKEWWNEFREKLWEAMLSEHKNNINNCKNIPQEELQIT</td></tr>
<tr><td>>PFIT_0730400_DBLtag2 | Plasmodium falciparum IT | erythrocyte binding antigen-175 | protein | length=1502</td><td>FADIRDIIGGTDYWNDLSNRKLVGKINTNSNYVHRNKQNDKLFRDEWWKVIKKDVWNVISWVFKDKTVCKEDDIENIPQFFR</td></tr>
<tr><td rowspan="2">PrCDC</td><td>>PrCDC1_EBA175 tag1</td><td>FLDYGHLAMGNDMDFGGYSTKAENKIQEVFKGVHGKISEHEIKNFRKKWWNEFREKLWEAMISEHKNNLSDCKNIPEEELQINQ</td></tr>
<tr><td>>PrCDC1_EBA175 tag2</td><td>FADIRDIIKGSDYWNDLSNRKLVGKINTNSKYVHKNKENDKLFRDEWWNVIKKDVWNVMSWVFKDKTVCKEDDIENIPQFFR</td></tr>
<tr><td rowspan="6">EBA-165</td><td rowspan="2">3D7</td><td>>PF3D7_0424300_DBLtag1 | Plasmodium falciparum 3D7 | erythrocyte binding antigen-165</td><td>YGDYGDIIKGTDMEGLGRSIKVEFEIDRIFKRDNKKSAENRKKWWEEISTHIWNAMIKEHKEKFPKDVKECSADKPLDEPQINR</td></tr>
<tr><td>>PF3D7_0424300_DBLtag2 | Plasmodium falciparum 3D7 | erythrocyte binding antigen-165</td><td>FDDYKNIILGKDMWRDPNSIKTENILKGNFEGIKANIVSMYPSYADLSLDEFRKHWWDQNKKQLWEAISCEFYKGNHTGVCLMEDDNDNQYLHWFR</td></tr>
<tr><td rowspan="2">IT</td><td>>PFIT_0422500_DBLtag1 | Plasmodium falciparum IT | erythrocyte binding antigen-165</td><td>YGDYGDIIKGTDMEGLGRSIKVEFEIDRIFKRDNKKSAENRKKWWEEISTHIWNAMIKEHKEKFPKDVKECSADKPLDEPQINR</td></tr>
<tr><td>>PFIT_0422500_DBLtag2 | Plasmodium falciparum IT | erythrocyte binding antigen-165</td><td>FDDYKNIILGKDMWRDPNSIKTENILKGNFEGIKANIVSMYPSYADLSLDEFRKHWWDQNKKQLWEAISCEFYKGNHTGVCLMEDDNDNQYLHWFR</td></tr>
<tr><td rowspan="2">PrCDC</td><td>>PRCDC EBA165 tag1</td><td>YGDYGDIIKGTDMEGLGRSIKVEFEIDRIFKRDNEKSAENRKKWWEEISSHIWNAMIKEHKENLPKDIKACSSDKPSDELQINR</td></tr>
<tr><td>>PRCDC EBA165 tag2</td><td>FDDYKNIILGKDMWKDANSMKTENILKGNFEGIKANIVRMYPGYADLSLDEFRKQWWDQNKNQLWEAISCEFYNGHHTGVCLMEDDDDNQYLH</td></tr>
<tr><td rowspan="6">EBA-140</td><td rowspan="2">3D7</td><td>>PF3D7_1301600_DBLtag1 | Plasmodium falciparum 3D7 | erythrocyte binding antigen-140 (EBA140) | protein | length=1210</td><td>FSDFRNSFIGDDMDFGGNTDRVKGYINKKFSDYYKEKNVEKLNNIKKEWWEKNKANLWNHMIVNHKGNISKECAIIPAEEPQINL</td></tr>
<tr><td>>PF3D7_1301600_DBLtag2 | Plasmodium falciparum 3D7 | erythrocyte binding antigen-140 (EBA140) | protein | length=1210</td><td>YADLADIIKGSDIIKDYYGKKMEENLNKVNKDKKRNEESLKIFREKWWDENKENVWKVMSAVLKNKETCKDYDKFQKIPQFLR</td></tr>
<tr><td rowspan="2">IT</td><td>>PFIT_1300800_DBLtag1 | Plasmodium falciparum IT | erythrocyte binding antigen-140 | protein | length=1210</td><td>FSDFRSSFIGDDMDFGGNTDRVKGYINKKFSDYYKEKNVEKLNNIKKEWWEKNKANLWNHMIVNHKGNISKECAIIPAEEPQINL</td></tr>
<tr><td>>PFIT_1300800_DBLtag2 | Plasmodium falciparum IT | erythrocyte binding antigen-140 | protein | length=1210</td><td>YADLADIIKGSDIIKDYYGKKMEENLNKVNKDKKRNEESLKIFREKWWDENKENVWKVMSAVLKNKETCKDYDKFQKIPQFLR</td></tr>
<tr><td rowspan="2">PrCDC</td><td>>PRCDC EBA140 tag1</td><td>FSDFRSLFIGDDMDFGGNTDRVKGYINMKFSDYYAEKNVEKLNNIKKEWWEKNKENLWNHMIVNHKENISNECATIPEEEPQINA</td></tr>
<tr><td>>PRCDC EBA140 tag2</td><td>YADFADIIKGSDIMKDYYGKNMEESLNKVNKDKKSNEESLKIFREKWWDENKENVWKVMSAVLKNKETCKDYDKFQKTPQFLR</td></tr>
<tr><td rowspan="5">EBA-181</td><td rowspan="2">IT</td><td>>PFIT_0101000_DBLtag1 | Plasmodium falciparum IT | erythrocyte binding antigen-181 | protein | length=1575</td><td>YLDYKDVIFGTDLDKNNISKLVEESLKRFFKKDSSVLNPTAWWRRYGTRLWKTMIQPYAHLGCRKPDENEPQINR</td></tr>
<tr><td>>PFIT_0101000_DBLtag2 | Plasmodium falciparum IT | erythrocyte binding antigen-181 | protein | length=1575</td><td>YVDYKDIVIGNDLWNDNNSIKVQNNLNLIFERNFGYKVGRNKLFKTIKELKNVWWILNRNKVWESMRCGIDEVDQRRKTCERIDELENMPQFFR</td></tr>
<tr><td rowspan="2">3D7</td><td>>PF3D7_0102500_DBLtag1 | Plasmodium falciparum 3D7 | erythrocyte binding antigen-181 (EBA181) | protein | length=1567</td><td>YLDYKDVIFGTDLDKNNISKLVEESLKRFFKKDSSVLNPTAWWRRYGTRLWKTMIQPYAHLGCRKPDENEPQINR</td></tr>
<tr><td>>PF3D7_0102500_DBLtag2 | Plasmodium falciparum 3D7 | erythrocyte binding antigen-181 (EBA181) | protein | length=1567</td><td>YVDYKDIVIGNDLWNDNNSIKVQNNLNLIFERNFGYKVGRNKLFKTIKELKNVWWILNRNKVWESMRCGIDEVDQRRKTCERIDELENMPQFFR</td></tr>
<tr><td>PrCDC</td><td>>PRCDC EBA181 DBLtag1</td><td>YLDYKDVIFGTDLDKNNISKIVEEALKRFFKKDSSILNPHAWWQRYGTRLWKYMIKPYEHLGCKKPDEGEPQINR</td></tr>
</table>

		>PRCDC EBA181 DBLtag2	YADYKDIVIGTDLWNDKNSIKAQNNLNAIFERNFGYKVGRNKLFKTIKDLRTVWWILNRDNIWESMKCGIIDVDRRGYSCVRMNELENMPQFFR
EBL-1	3D7	>PF3D7_1371600_DBLtag1 | Plasmodium falciparum 3D7 | erythrocyte binding like protein 1	FLDYGDIIIGRDLIYKNNTDYIKEQFKKIFNNEYNNNELNDELNNELNDEKNIKLRKEWWEKYKEDIWEEMTKEHNDKFIEKCKYFAKDEPQIVR
		>PF3D7_1371600_DBLtag2 | Plasmodium falciparum 3D7 | erythrocyte binding like protein 1	YADIGDIVRGLDVWRDINTNKLSEKFQKIFMGGGNSRKKQNDNNERNKWWEKQRNLIWSSMVKHIPKGKTCKRHNNFEKIPQFLR
	IT	>PFIT_0731900_DBLtag1 | Plasmodium falciparum IT | erythrocyte binding like protein 1	FLDYGDIIIGRDLIYKNNTDYIKEQFKKIFNNEYNNNELNDELNNELNDEKNIKLRKEWWEKYKEDIWEEMTKEHNDKFIEKCKYFAKDEPQIVR
		>PFIT_0731900_DBLtag2 | Plasmodium falciparum IT | erythrocyte binding like protein 1	YADIGDIVRGLDVWRDINTNKLSEKFQKIFMGGGNSRKKQNDNNERNKWWEKQRNLIWSSMVKHIPKGKTCKRHNNFEKIPQFLR
P. falciparum DBLMSP	3D7	>PF3D7_1035700 | Plasmodium falciparum 3D7 | duffy binding-like merozoite surface protein (DBLMSP) | protein | length=697	FSDMGDIIKGTDLIDYQITKNINRALDKILRNETSNDKIKKRVDWWEANKSAFWDAFMCGYKVHIGNKPCPEHDNMDRIPQYLR
	IT	>PFIT_1035100 | Plasmodium falciparum IT | duffy binding-like merozoite surface protein | protein | length=697	FSDMGDIIKGTDLIDYQITKNINRALDKILGNETSNDKIKKRVDWWEANKSAFWDAFMCGYKVHIGNKPCPEHDNMDRIPQYLR
	PrCDC	>PRCDC_1035000 | Plasmodium reichenowi PRCDC | duffy binding-like merozoite surface protein MSP3.4 MSPDBL1	FSDMGDIIKGTDLIDYQITKNINRALDKILGNERSNAEIKNRVDWWEANKSELWDALMCGYKIHIGNKACPEHDNMDRIPQYLR
P. falciparum DBLMSP2	3D7	>PF3D7_1036300 | Plasmodium falciparum 3D7 | merozoite surface protein (DBLMSP2) | protein | length=762	FADIGNIIRGDDMMDTPTSKETITYLEKVLKIYNENNDKPKDAKKWWTENRHHVWEAMMCGYQSAQKDNQCTGYGNIDDIPQFLR
	IT	>PFIT_1035500 | Plasmodium falciparum IT | merozoite surface protein (MSP3.8) | protein | length=767	FADIGNIVQGNDMIDTPTSNKTKTYLEEVLGKHYKNVNDPKDAKTWWIQNKHRVWDAMMCGYQYEKKDNKCTGYGNIYDIPQFLR
	PrCDC	>PRCDC_1035400 | Plasmodium reichenowi PRCDC | merozoite surface protein PSEUDOGENE MSP3.8 MSPDBL2	FADIGNIIKGDDILDTPTSNKTKTYLEEVLELHNKNDEAKDAKKWWTENRHHISKAMMYGYQNEKKDNECTVYGNIDDIPQFLR

Supplementary Table 4. List of PCR primers used for *Laverania* species identification.

gene	1st Round PCR	2nd Round PCR
**eba165**	PfrEBA165F3: 5'-ATGTAATGAGAAAGACCTTGAAGTTG-3'	PfrEBA165F4: 5-'CTCTGGGTTCCTCTTTTCTTCTA-3'
	PfrEBA165R24: 5'-CATAAGCCCATTCCATTAACCATC-3'	PfrEBA165R25: 5'-TACCAAGTCCTTCCATGTCAGTA-3'
**p47**	Pfs47F449: 5'-GTAGATGTGATAATAGTAAAACGG-3'	Pfs47D2F1: 5'-TATCCCAGGACAAGATAAAATAT-3'
	Pfs47R1: 5'-AATGTATTGGAAAACATTCCATATAC-3'	Pfs47R3: 5'-CAAGTTCATTCATATGYTAAMATACAT-3'
**p48/45**	P48/45F1: 5'-TGTAATTTTTCAAATGAAGGTGTTC-3'	P48/45F2: 5'-AGCCRGATAYGCGTGAACGTAGGTC-3'
	P48/45R1: 5'-ATTTTATCATCTCCTTCARCATCTT-3'	P48/45R2: 5'-TGGATGGTTCAAGTTCTTCTGATTC-3'
**RH5**	PfrRH5F5: 5'-CRAAGAATCAAGAAAATAATCTGAC-3'	PfrRH5F6: 5'-TTGTTTATKCCTTCTCATAATKCTT-3'
	PfrRH5R3: 5'-GATGSTTTGTYTAATTAGRGTATATGA-3'	PfrRH5R4: 5'-CCATGTTTTGTCATTTCATTGYGTAAG-3'
**eba 175**[a]	EBA175sf2: 5′-CATCTTGCTATGGGAAATGATATGG-3′	EBA175sf3: 5′-GAAGGAATGTATTGATCCATGTATG-3′
	EBA175sr2: 5′-TTRCTCAAATCATTCCAATAATCAG-3′	EBA175sr3: 5′-TATATTCTATCAATGTTTCCAAGAC-3′

[a] See Supplementary Ref [4].

Supplementary References

1. Rask, T. S., Hansen, D. A., Theander, T. G., Gorm Pedersen, A. & Lavstsen, T. *Plasmodium falciparum* erythrocyte membrane protein 1; diversity in seven genomes – divide and conquer. *PLoS Comput. Biol.* **6,** e1000933 (2010).
2. Bull, P. C. *et al. Plasmodium falciparum* variant surface antigen expression patterns during malaria. *PLoS Pathog.* **1,** e26 (2005).
3. Liu, W. *et al.* African origin of the malaria parasite *Plasmodium vivax*. *Nature Comm.* **5,** 1–10 (2014).
4. Wanaguru, M., Liu, W., Hahn, B. H., Rayner, J. C., Wright, G. J., RH5-Basigin interaction plays a major role in the host tropism of *Plasmodium falciparum*. Proc. Natl. Acad. Sci. USA, **110**, (2013)
